# Prefecture-Level Mean Elevation, Sleep Duration, and Depressive Symptoms Among Middle-Aged and Older Adults in China: A Cross-Sectional CHARLS Study

**DOI:** 10.3390/healthcare14203544

**Published:** 2026-10-21

**Authors:** Zhecheng Liu, Changxu Chen, Chuankai Shi, Yuan Gao, Yiyang Li, Xiaoli Xu, Hanxing Li, Siyu Xia, Shiyu Li, Jiangwei Liu

**Affiliations:** 1 Department of Graduate School, Xinjiang Medical University, Urumqi 830054, China; liuzhecheng2001@163.com (Z.L.); sdlzccx@163.com (C.C.); chuankai.shi@foxmail.com (C.S.); 2 Key Laboratory of Special Environmental Medicine of Xinjiang, General Hospital of Xinjiang Military Command, Urumqi 830000, China; 3 Xinjiang Medical University, Urumqi 830099, Chinalsy1122702@foxmail.com (S.L.)

**Keywords:** residential altitude, sleep duration, depressive symptoms, CHARLS, spatial epidemiology

## Abstract

**Highlights:**

**What are the main findings?**

- Mental health assessments among middle-aged and older adults may benefit from considering regional context and sleep duration patterns.
- Sleep duration showed observational associations with depressive symptoms across different geographic settings. Geographic differences in depressive symptoms should be interpreted considering both environmental exposure and regional socioeconomic context.

**What are the implications of the main findings?**

- Nonlinear analyses revealed complex patterns between altitude, sleep duration, and depressive symptoms rather than simple linear relationships.
- Sleep duration was associated with different patterns of depressive symptom scores across altitude groups, with short sleepers showing higher predicted CESD-10 scores. Prefecture-level mean elevation was positively associated with depressive symptom scores in crude models, but this association was attenuated after adjustment for individual characteristics, sleep duration, and province-level factors.

**Abstract:**

**Background:** Prefecture-level mean elevation, as an area-level proxy of residential altitude, has been associated with sleep disturbance and depressive symptoms, but it may also reflect broader regional differences in social conditions. The roles of sleep duration and regional context remain uncertain among long-term community residents. **Objectives:** To examine these associations among adults aged 45 years or older in China. **Methods:** This cross-sectional study linked nationally representative China Health and Retirement Longitudinal Study Wave 4 data from 15,562 adults aged 45 years or older with prefecture-level mean elevation. Linear regression with province fixed effects assessed sleep duration and CESD-10 scores. Altitude-by-sleep interactions, generalized additive models, restricted cubic splines, and exploratory bootstrap mediation analyses were conducted. **Results:** Moderate and high altitude were associated with higher CESD-10 scores in unadjusted models, but not after adjustment for sleep category, individual covariates, and province fixed effects. They were associated with 0.280 and 0.363 h longer self-reported sleep. The altitude-by-sleep interaction was significant, and predicted CESD-10 scores were highest among short sleepers across altitude groups. Nonlinear patterns were observed, whereas indirect effects through continuous sleep duration were not significant. **Conclusions:** The apparent association between higher altitude and depressive symptoms was not robust after regional adjustment. Sleep duration did not show evidence of mediation, although sleep category was associated with differences in the observed altitude–depressive symptom pattern.

## 1. Introduction

Sleep is a central behavioral determinant of emotional regulation and psychological well-being. Prospective evidence indicates that short sleep and insomnia are associated with a higher subsequent risk of depressive symptoms and depression, while deviations from an adequate sleep duration may be particularly relevant in middle-aged and older populations [1,2,3,4]. Sleep is also shaped by the physical and social environment in which people live. Understanding environmental influences on sleep may therefore help clarify geographic variation in mental health.

High-altitude environments are characterized by lower barometric pressure and reduced oxygen availability, together with colder temperatures and marked geographic isolation in many regions. Experimental and clinical studies of acute altitude exposure have documented periodic breathing, nocturnal oxygen desaturation, and sleep fragmentation [5,6]. Recent studies further showed that acute high-altitude exposure may induce measurable sleep disturbance among previously low-altitude residents [7,8,9,10]. These findings provide a plausible physiological basis for an altitude-sleep relationship. However, most such evidence comes from lowlanders shortly after ascent. Long-term high-altitude residents may differ from recently exposed lowlanders because prolonged exposure may involve physiological acclimatization, behavioral adaptation, and established environmental routines. A recent review has similarly emphasized that sleep-related respiratory responses differ across acclimatizing lowlanders and adapted highlanders [10]. Therefore, findings from acute exposure studies cannot be directly extrapolated to long-term community residents.

Population-based studies have reported associations between residential altitude and depressive symptoms, although effect estimates vary across geographic settings and study populations [11,12,13,14,15,16]. Evidence specifically from long-term high-altitude residents is heterogeneous. A community-based study of older highlanders in Ladakh and Qinghai reported a relatively low prevalence of clinically assessed depression despite residence at approximately 3700–3800 m [15], whereas a comparative study of older nursing-home residents reported a substantially higher prevalence of depression in the high-altitude group than in the low-altitude group [16]. Some studies have also investigated associations between altitude and anxiety-related outcomes [17]. Ecological studies have reported associations between altitude and suicide risk, although regional socioeconomic differences may contribute to these findings [18,19,20]. Several mechanisms have been proposed, including the possibility that hypobaric hypoxia may influence mood through sleep disruption, neuroendocrine stress, inflammation, or altered neurotransmitter metabolism [18]. At the same time, an altitude measure is not an isolated biological dose. Higher-altitude areas often differ from lower-altitude areas in economic development, health-care accessibility, population density, urban-rural composition, transportation, climate, culture, and social support. Consequently, an observed altitude-mental-health association may reflect both natural environmental exposure and contextual regional differences [21].

This distinction is especially important in China, where elevation follows a strong west-to-east spatial gradient and is closely intertwined with regional patterns of development and service availability. Conventional individual-level covariate adjustment may not fully address this spatial confounding. Area fixed-effects approaches can help control shared regional characteristics, although they cannot eliminate all unmeasured variation within regions. Examining how altitude coefficients change after regional adjustment may therefore be as informative as estimating the crude altitude association itself.

Sleep may occupy more than one position in this framework. First, it may act as an intermediate pathway if altitude-related environmental conditions alter sleep, which in turn influences depressive symptoms. Second, sleep duration may be associated with heterogeneity in the altitude–depression relationship. Experimental and meta-analytic evidence indicates that sleep loss increases negative mood and impairs adaptive emotion regulation, which may increase vulnerability to environmental or psychosocial stressors [22]. Mediation and interaction address different questions and should not be treated as interchangeable. A cross-sectional analysis cannot establish temporal mediation, but it can evaluate whether the observed data are compatible with a simple indirect pathway and whether altitude-sleep combinations show non-additive patterns.

Important gaps therefore remain. National evidence among long-term community-dwelling middle-aged and older adults is limited; sleep duration, mediation, and effect modification have rarely been evaluated together; and categorical altitude comparisons may conceal nonlinear exposure-response patterns. Moreover, because geographic altitude may proxy broader regional context, analyses that do not account for area-level background can overstate an independent altitude effect.

Using CHARLS Wave 4 data linked to prefecture-level mean elevation, our primary analyses examined whether elevation was associated with self-reported sleep duration and CESD-10 scores after individual and regional adjustment, and whether CESD-10 patterns differed across combinations of elevation and sleep-duration categories. Mediation, nonlinear exposure–response, subgroup, and sensitivity analyses were treated as exploratory analyses intended to characterize potential statistical pathways, assess heterogeneity, and evaluate the robustness of the primary findings. We hypothesized that short sleep would be associated with higher CESD-10 scores and that the association between prefecture-level mean elevation and depressive symptoms would be attenuated after regional adjustment.

## 2. Methods

### 2.1. Research Design and Data Sources

We conducted a cross-sectional secondary analysis of CHARLS Wave 4 (2018), a nationally representative survey designed to study Chinese adults aged 45 years or older [23,24]. Individual-level survey data were linked to prefecture-level mean elevation, which was treated as an area-level proxy of residential altitude.

Sampling weights, stratification variables, and primary sampling unit identifiers were not incorporated into the regression analyses because the primary objective was to evaluate conditional associations within the analytical sample rather than to estimate population prevalence. Accordingly, the findings should be interpreted as associations within the analytical sample rather than as weighted national population estimates.

The CHARLS protocol was approved by the Institutional Review Board of Peking University (IRB00001052-11015), and written informed consent was obtained from all participants [24]. The present secondary analysis was conducted in accordance with the ethical principles of the Declaration of Helsinki. No additional pilot study or questionnaire modification was conducted for the present secondary analysis; all study variables were derived from the standardized CHARLS Wave 4 dataset.

### 2.2. Prefecture-Level Elevation and Spatial Linkage

**Figure 1:**
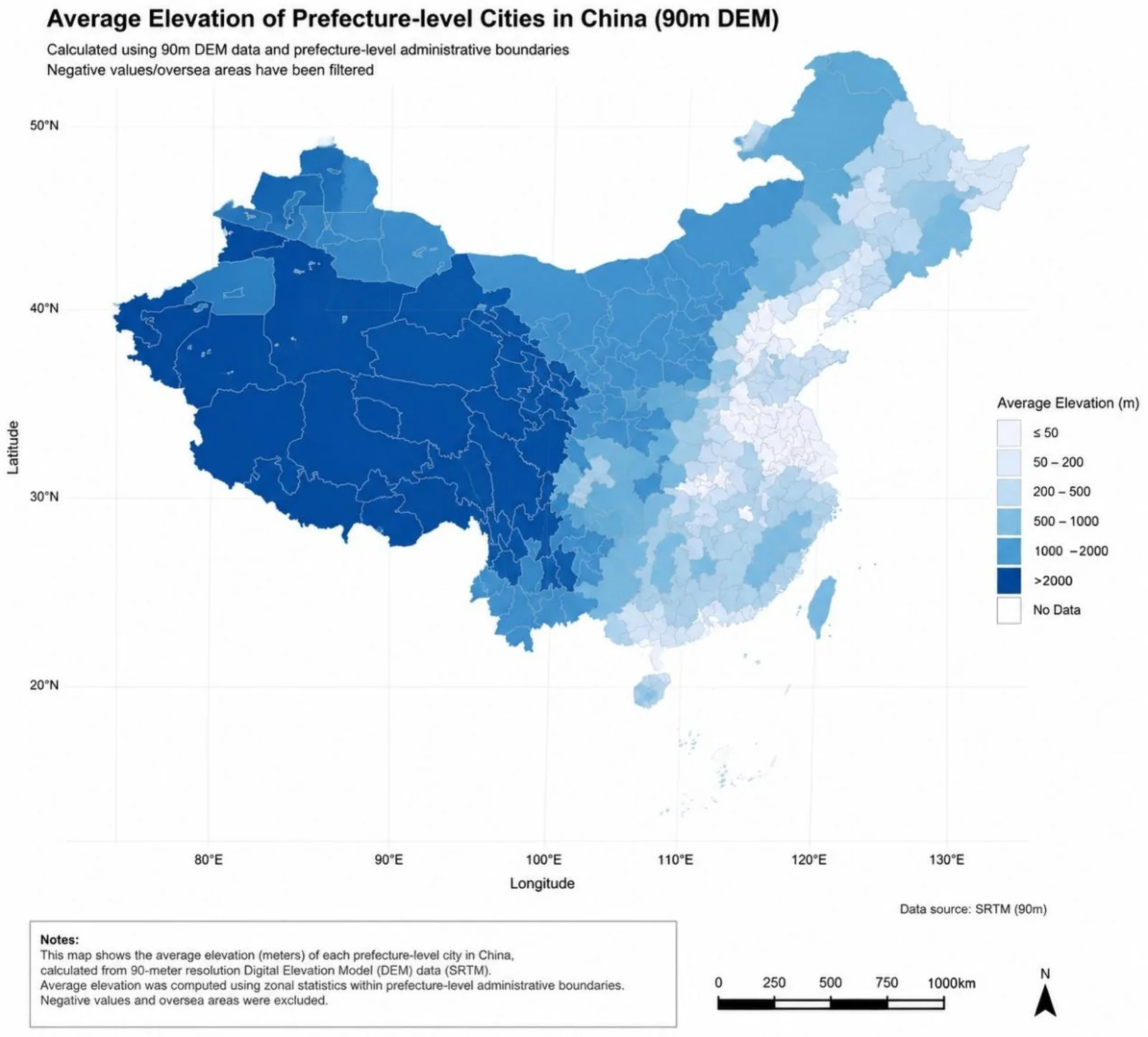
Spatial distribution of prefecture-level mean elevation in China.

Digital elevation data were obtained from the 90-m Shuttle Radar Topography Mission (SRTM) digital elevation model available through the China Geospatial Data Cloud; the SRTM elevation data were acquired in 2000 [25]. Prefecture-level administrative boundaries were obtained from the National Geographic Information Public Service Platform. Because individual residential coordinates were unavailable, prefecture-level mean elevation was used as an area-level proxy to characterize the overall topographic context of each prefecture. Mean elevation was calculated using zonal statistics and linked to participants after standardization of prefecture names [21,25]. To address potential uncertainty related to historical administrative changes or ambiguous prefecture names, a sensitivity analysis was additionally performed after excluding participants linked to Chaohu and Xiangfan. Accordingly, prefecture-level mean elevation should be interpreted as an area-level proxy of residential elevation rather than the precise elevation of an individual participant’s home; its spatial distribution is shown in Figure 1.

Prefecture-level mean elevation was calculated from digital elevation model data using prefecture-level administrative boundaries. The figure illustrates the spatial distribution of the area-level altitude exposure used in this study. Elevation values represent prefecture-level mean elevation and should not be interpreted as precise residential altitude for individual participants.

### 2.3. Study Population and Sample Selection

Participants were eligible if they were aged ≥ 45 years, reported a total daily sleep duration of ≥2 and ≤12 h, had valid CESD-10 and province information, had complete data for the prespecified covariates, and could be successfully linked to prefecture-level mean elevation. Sleep durations of <2 h or >12 h were excluded as extreme self-reported values because they may reflect irregular sleep patterns, reporting errors, or underlying health conditions rather than habitual sleep duration, which could introduce bias in analyses treating sleep duration as a continuous variable [26].

No additional exclusion criterion was applied solely on the basis of self-reported mental illness or cognitive impairment; participants were retained if they otherwise met the eligibility criteria and had the required study variables.

**Figure 2:**
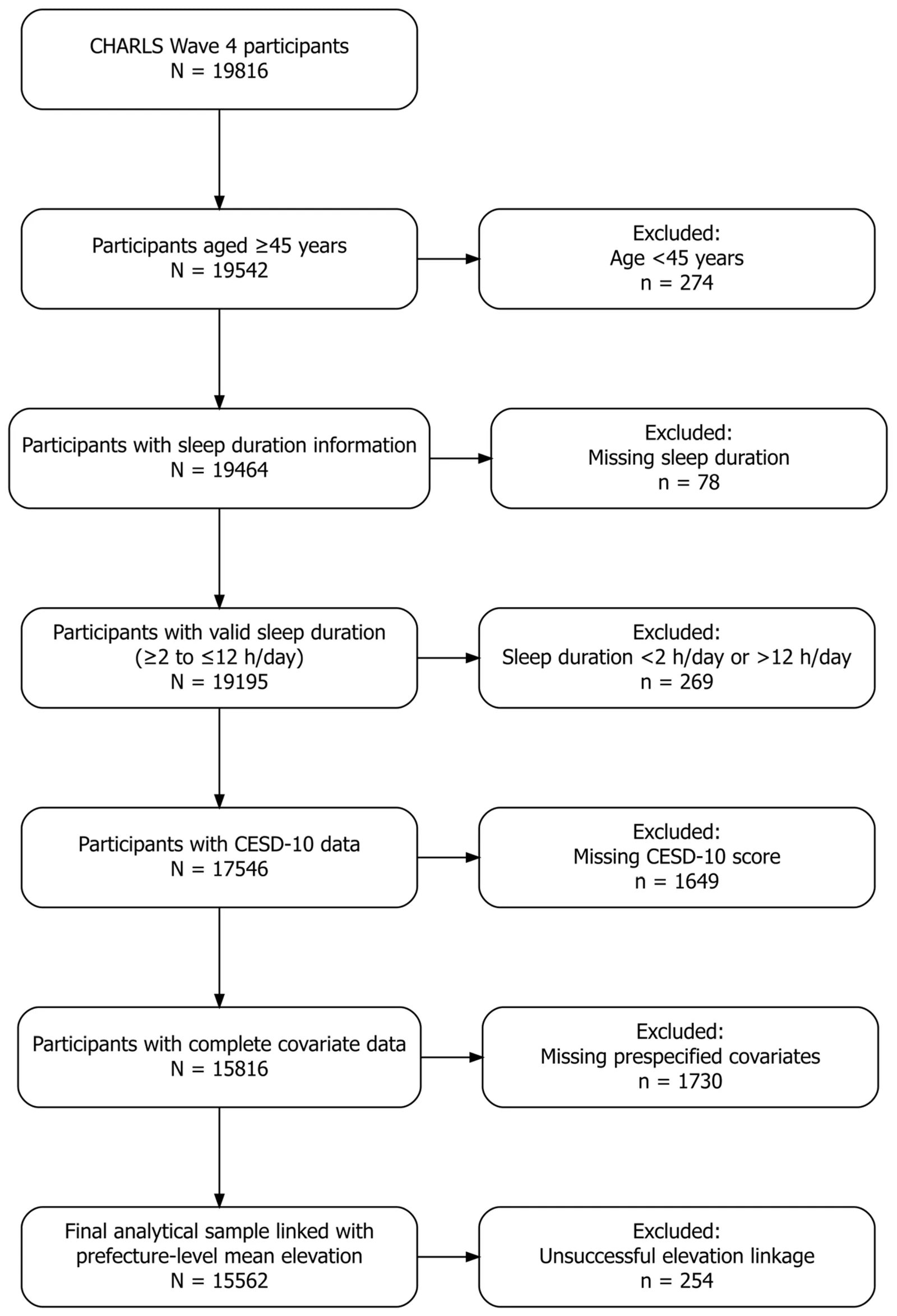
Flowchart of sample selection.

Complete-case analysis was used for the required outcome, exposure, and covariate data. After sequential exclusions, 15,562 participants remained in the analytical sample (Figure 2). Because exclusions due to missing information or unsuccessful geographic linkage may have introduced selection bias, this possibility was considered when interpreting the findings.

The analytical sample was derived from CHARLS Wave 4 participants. Exclusions were applied sequentially for age, missing or invalid sleep duration, missing CESD-10 score, incomplete covariates, and unsuccessful linkage to prefecture-level mean elevation. The final analytical sample included 15,562 participants.

### 2.4. Variables

#### 2.4.1. Prefecture-Level Mean Elevation

The primary exposure was prefecture-level mean elevation, categorized as low (<1000 m), moderate (1000–2000 m), or high (>2000 m), with the low-elevation group as the reference. These thresholds were selected as pragmatic epidemiological contrasts based on previous altitude-health studies rather than as clinical or physiological cut points [11,26,27,28]. Sensitivity analyses used an alternative categorization of <1000 m, 1000–1500 m, and ≥1500 m. Prefecture-level mean elevation was additionally modeled as a continuous exposure in exploratory nonlinear analyses to reduce dependence on predefined categorical thresholds.

#### 2.4.2. Definition of Sleep Duration

Self-reported total daily sleep duration, including nighttime sleep and daytime naps, was analyzed continuously and categorized as short (<6 h), normal (6–8 h), or long (>8 h) sleep [29,30]. Sleep duration was analyzed continuously in the nonlinear and mediation analyses and categorically in the interaction analysis. Short sleep was the reference category in the interaction model.

#### 2.4.3. Depressive Symptoms

Depressive symptoms were assessed using the 10-item Center for Epidemiologic Studies Depression Scale (CESD-10), which has demonstrated acceptable reliability and validity among older adults and Chinese populations [31,32,33,34,35]. Among 13,870 participants in the analytic cohort with complete responses to all 10 CESD items, the scale demonstrated good internal consistency (Cronbach’s α = 0.802). Total scores range from 0 to 30, with higher scores indicating greater depressive symptom severity. CESD-10 score was analyzed as a continuous outcome; regression coefficients therefore represent mean differences in depressive symptom scores rather than the risk or diagnosis of clinical depression.

### 2.5. Covariates

Covariates were prespecified on the basis of previous literature and the conceptual framework as potential individual-level confounders of the associations among geographic context, sleep duration, and depressive symptoms [11,36,37,38]. These included age, sex, education, smoking, alcohol drinking, chronic disease, pension, medical insurance, urban or rural residence, regular exercise, and marital status. Age was modeled continuously, whereas the remaining covariates were categorical. Province fixed effects were incorporated into regionally adjusted models to account for contextual characteristics shared within provinces while retaining within-province variation in prefecture-level mean elevation [21]. Province fixed effects and sleep duration were introduced separately in the sequential models to distinguish attenuation associated with regional adjustment from that associated with sleep adjustment. Prefecture fixed effects were not included because the exposure was assigned at the prefecture level and prefecture fixed effects would absorb the between-prefecture elevation contrast of interest. A directed acyclic graph summarizing the assumed relationships among prefecture-level mean elevation, sleep duration, depressive symptoms, and covariates is provided in Figure S1.

### 2.6. Descriptive Statistics

Baseline characteristics were summarized according to prefecture-level mean elevation category as mean (SD) for continuous variables and n (%) for categorical variables. Continuous variables were compared using Kruskal–Wallis tests and categorical variables using Pearson’s chi-squared tests. Given the large analytical sample, baseline *p* values were interpreted descriptively rather than as evidence of clinically meaningful between-group differences.

### 2.7. Regression Analysis of Prefecture-Level Mean Elevation and Sleep Duration

A linear regression model with province fixed effects was used to estimate differences in continuous sleep duration across prefecture-level mean elevation categories, adjusting for the prespecified covariates. Standard errors were clustered at the prefecture level, and the low-elevation group was used as the reference.

### 2.8. Association Between Prefecture-Level Mean Elevation and Depressive Symptoms

Linear regression models were used to examine the association between prefecture-level mean elevation category and CESD-10 score. Model 1 was unadjusted. Model 2 adjusted for age, sex, and education. Model 3 further adjusted for the remaining prespecified individual-level covariates. To distinguish regional adjustment from sleep adjustment, Model 4a additionally included province fixed effects, Model 4b additionally included sleep-duration category, and Model 4c included both sleep-duration category and province fixed effects.

### 2.9. Modeling of the Elevation–Sleep Duration Interaction

A fully adjusted model included prefecture-level mean elevation category, sleep-duration category, and their interaction terms, together with the prespecified individual-level covariates and province fixed effects. Low elevation and short sleep constituted the joint reference category. The overall prefecture-level mean elevation-by-sleep interaction was evaluated using a joint Wald test of the interaction terms. Marginal-standardized predicted CESD-10 scores were calculated for each elevation-by-sleep combination by standardizing model predictions over the covariate distribution of the analytical sample. These estimates were used to characterize the adjusted interaction pattern and should not be interpreted as raw group means or as evidence of causal or biological interaction.

### 2.10. Nonlinear Analyses

Generalized additive models (GAMs) were used to examine potentially nonlinear associations of continuous sleep duration and continuous prefecture-level mean elevation with CESD-10 score [39,40]. Penalized smooth terms with a basis dimension of k = 5 were fitted, with smoothing parameters estimated using restricted maximum likelihood (REML). Adjusted predicted curves with 95% confidence intervals were generated, and rug plots were used to display the distribution of observed exposure values.

#### 2.10.1. Sleep Duration

The sleep-duration GAM included a smooth term for continuous sleep duration and adjusted for prefecture-level mean elevation category and the prespecified individual-level covariates.

#### 2.10.2. Continuous Prefecture-Level Mean Elevation

The elevation GAM included a smooth term for continuous prefecture-level mean elevation and adjusted for sleep-duration category and the prespecified individual-level covariates.

### 2.11. Mediation Analysis

Exploratory mediation analyses were conducted separately for moderate versus low elevation and high versus low elevation [41]. Prefecture-level mean elevation category was treated as the exposure, continuous sleep duration as the mediator, and CESD-10 score as the outcome. Both the mediator and outcome models adjusted for the prespecified individual-level covariates and province fixed effects. Nonparametric bootstrap inference with 2000 replications was used to estimate the average causal mediation effect (ACME), average direct effect (ADE), total effect, proportion mediated, and corresponding 95% confidence intervals [42]. Continuous sleep duration was used in the mediation analysis to preserve information when evaluating a potential statistical indirect pathway, whereas categorical sleep duration was used in the interaction analysis to facilitate interpretation of joint exposure patterns. Because exposure, sleep duration, and depressive symptoms were assessed cross-sectionally, the mediation analysis was considered exploratory and was not interpreted as evidence of a causal or temporally ordered mediation pathway.

### 2.12. Subgroup Analysis

Exploratory analyses were stratified by sex, age (<60 vs. ≥60 years), and education (primary school or less vs junior high school or above). Within each subgroup, models adjusted for the remaining prespecified covariates, sleep-duration category, and province fixed effects. Formal interaction tests comparing estimates across subgroup strata were not performed, and no adjustment for multiple comparisons was applied. Accordingly, subgroup findings were considered exploratory and were interpreted descriptively rather than as confirmatory evidence of effect modification.

### 2.13. Sensitivity Analyses

Sensitivity analyses evaluated the robustness of the primary findings by (1) reclassifying prefecture-level mean elevation using an alternative threshold of 1500 m; (2) examining an elevation-by-regular-exercise interaction; and (3) repeating the fully adjusted analysis after excluding participants linked to Chaohu and Xiangfan because of potential uncertainty in historical or ambiguous place-name matching.

### 2.14. Statistical Analysis

All analyses were conducted in R and reported in accordance with the STROBE statement [43]. Because prefecture-level mean elevation was assigned at the prefecture level and multiple participants therefore shared the same area-level exposure value, prefecture-clustered robust standard errors were used in the main regression and elevation-by-sleep interaction models to account for within-prefecture correlation. Regression results are reported as β coefficients with 95% confidence intervals (CIs) and two-sided *p* values. Statistical significance for the primary analyses was defined as *p* < 0.05. The overall elevation-by-sleep interaction was assessed using a joint Wald test. GAM results were characterized using estimated smooth functions, effective degrees of freedom, approximate F statistics, corresponding *p* values, and adjusted predicted curves with 95% CIs. Because the mediation, nonlinear, subgroup, and sensitivity analyses were treated as exploratory, no formal adjustment for multiple testing was applied. Findings from these secondary analyses were therefore interpreted cautiously in conjunction with their effect estimates and confidence intervals.

## 3. Results

### 3.1. Baseline Characteristics of the Study Population

The analysis included 15,562 participants: 12,453 at low, 2112 at moderate, and 997 at high altitude. Mean age was 61.7 (SD 9.5) years, and 50% were women (Table 1).

Compared with the low-altitude group, participants at higher altitude were younger, more often rural, less educated, and less likely to have a pension. Mean CESD-10 scores were 7.9, 9.3, and 9.9 across low, moderate, and high altitude, respectively.

Mean sleep duration differed little across groups (6.2, 6.3, and 6.3 h; *p* = 0.065), although the distribution of sleep categories varied (*p* < 0.001).

### 3.2. Association Between Prefecture-Level Mean Elevation and Sleep Duration

Continuous sleep duration showed little unadjusted variation by altitude (Figure S2). In the adjusted province fixed-effects model, moderate and high elevation were associated with 0.280 h (95% CI, −0.012 to 0.571; *p* = 0.060) and 0.363 h (95% CI, −0.109 to 0.834; *p* = 0.130) longer sleep, respectively, compared with low elevation; neither estimate reached statistical significance (Table S1). The point estimates were modest, and both confidence intervals included the null.

### 3.3. Prefecture-Level Mean Elevation and Depressive Symptoms Across Sequential Models

In the unadjusted model, moderate and high elevation were associated with higher CESD-10 scores (moderate: β = 1.409, 95% CI 0.376 to 2.443, *p* = 0.008; high: β = 1.997, 95% CI 0.859 to 3.136, *p* < 0.001). Estimates remained positive after demographic and multivariable adjustment in Models 2 and 3 (Table 2). After province fixed effects were added to Model 3 (Model 4a), the elevation coefficients were markedly attenuated and were no longer statistically significant (moderate: β = −0.368, *p* = 0.527; high: β = −0.369, *p* = 0.575). In contrast, adding sleep-duration category without province fixed effects (Model 4b) produced little attenuation, and both associations remained statistically significant (moderate: β = 1.153, *p* = 0.006; high: β = 1.811, *p* < 0.001). In Model 4c, which included both sleep-duration category and province fixed effects, neither elevation association was statistically significant (moderate: β = −0.288, *p* = 0.574; high: β = −0.174, *p* = 0.751; Table 2). The fully adjusted altitude estimates are also shown graphically in Supplementary Figure S4.

### 3.4. Prefecture-Level Mean Elevation–Sleep Interaction

**Figure 3:**
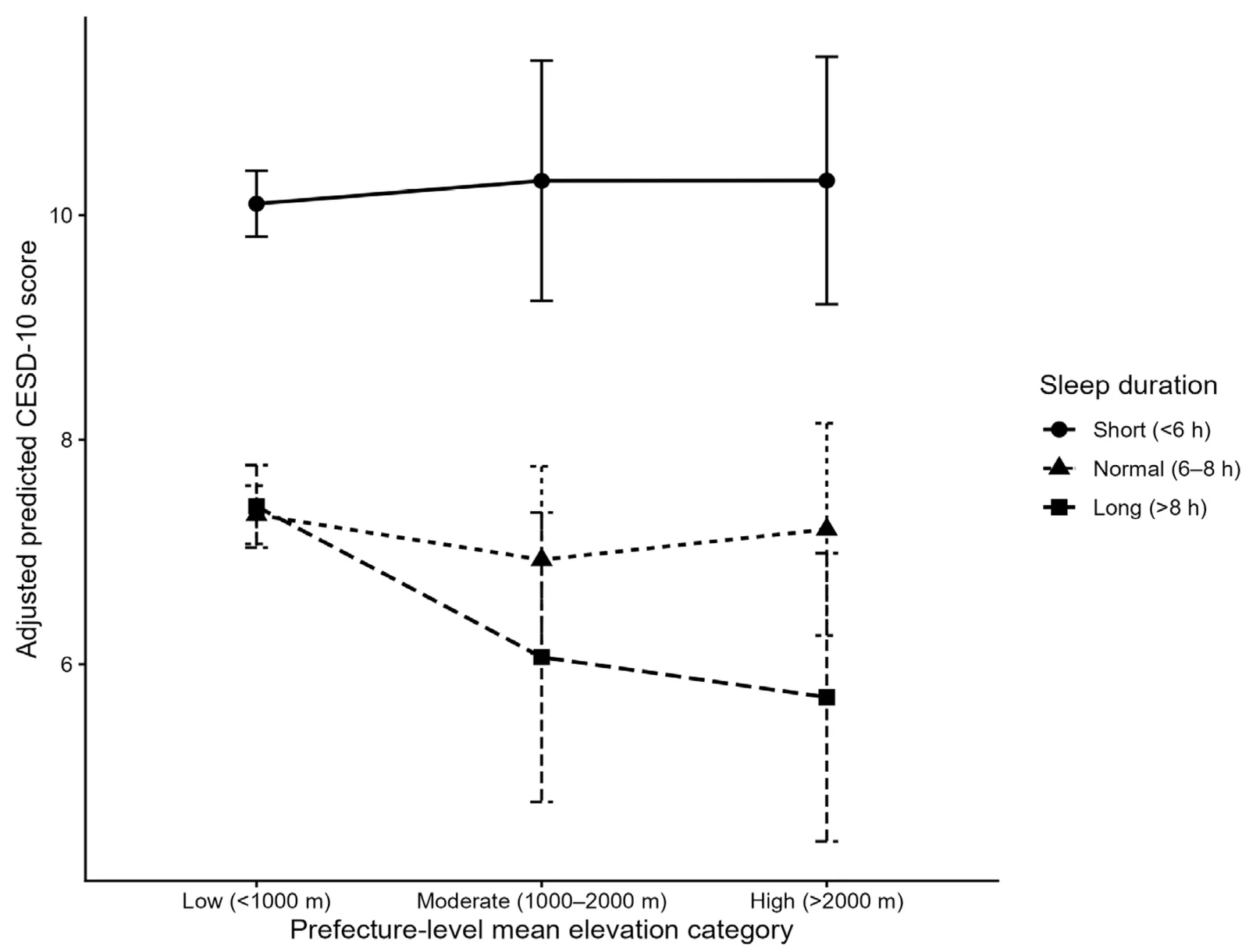
Marginal-standardized predicted CESD-10 scores across prefecture-level mean elevation and sleep-duration categories. Predictions were obtained from the fully adjusted prefecture-level mean elevation × sleep-duration interaction model and represent model-standardized estimates rather than raw group means.

The overall prefecture-level mean elevation-by-sleep interaction was statistically significant (joint Wald χ^2^ = 17.022, df = 4, *p* = 0.002; Table 3) The interaction terms for moderate elevation × long sleep (β = −1.549, 95% CI −2.671 to −0.427, *p* = 0.007) and high elevation × long sleep (β = −1.908, 95% CI −3.444 to −0.372, *p* = 0.015) were statistically significant, whereas the interactions involving normal sleep were not (*p* = 0.107 and *p* = 0.505, respectively). Marginal-standardized CESD-10 scores among short sleepers were 10.1, 10.3, and 10.3 at low, moderate, and high altitude, respectively. Corresponding scores were 7.3, 6.9, and 7.2 for normal sleep and 7.4, 6.1, and 5.7 for long sleep (Figure 3; Figure S3).

These predictions describe adjusted patterns and are not raw group means.

### 3.5. Nonlinear Association Between Sleep Duration and Depressive Symptoms

**Figure 4:**
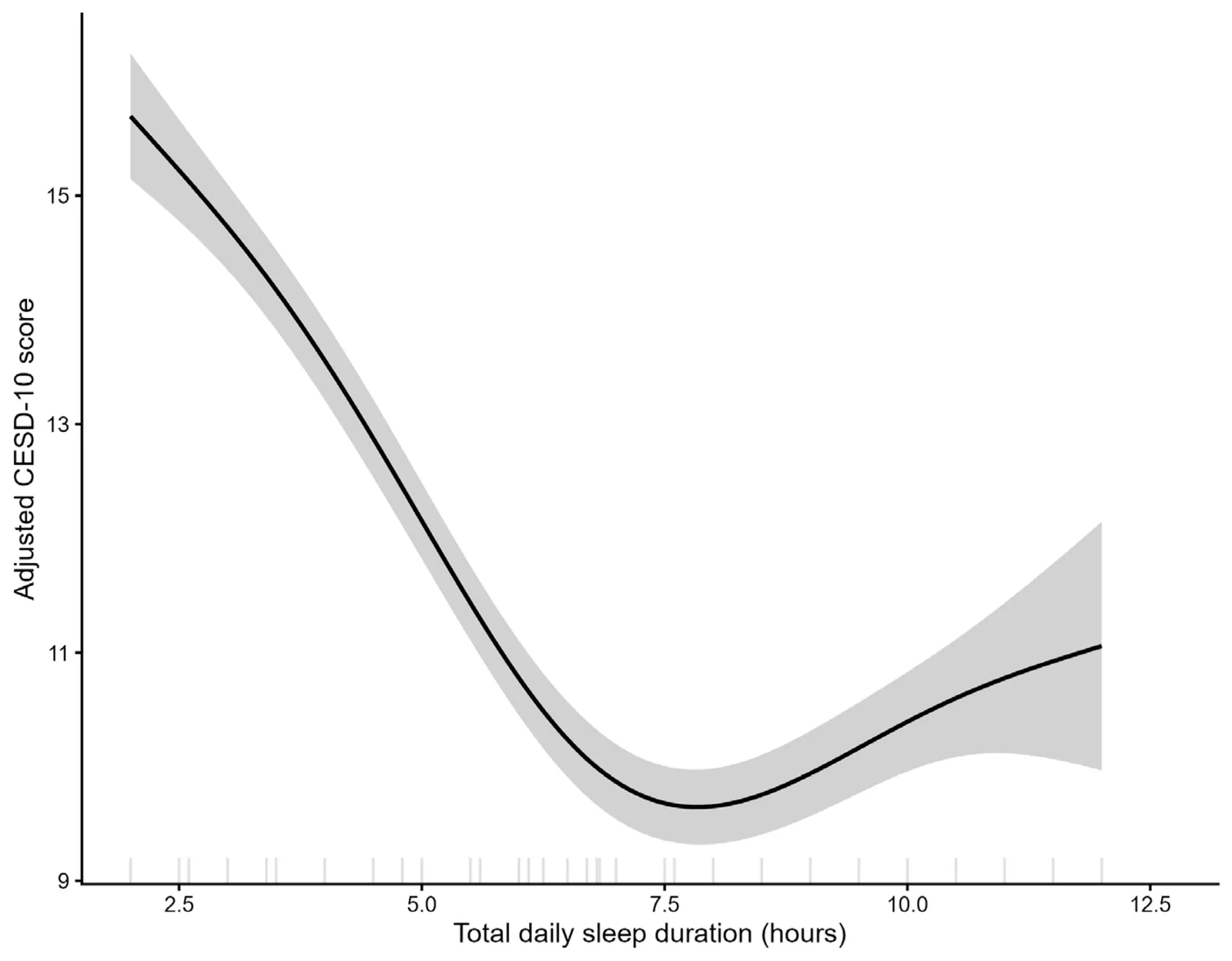
Nonlinear association between continuous sleep duration and CESD-10 scores estimated using generalized additive models. The curve was estimated using a generalized additive model adjusted for prefecture-level mean elevation category and prespecified individual-level covariates. The shaded area represents the 95% confidence interval.

The adjusted GAM showed a nonlinear association between total daily sleep duration and CESD-10 score (edf = 3.848, F = 296.762, *p* < 0.001; Figure 4). A formal comparison of the linear and smooth specifications also supported nonlinearity (F = 89.756, *p* < 0.001). Predicted scores declined as sleep increased, were lowest at approximately 7–9 h, and rose modestly at longer durations; uncertainty widened at the upper end.

### 3.6. Nonlinear Association Between Prefecture-Level Mean Elevation and Depressive Symptoms

**Figure 5:**
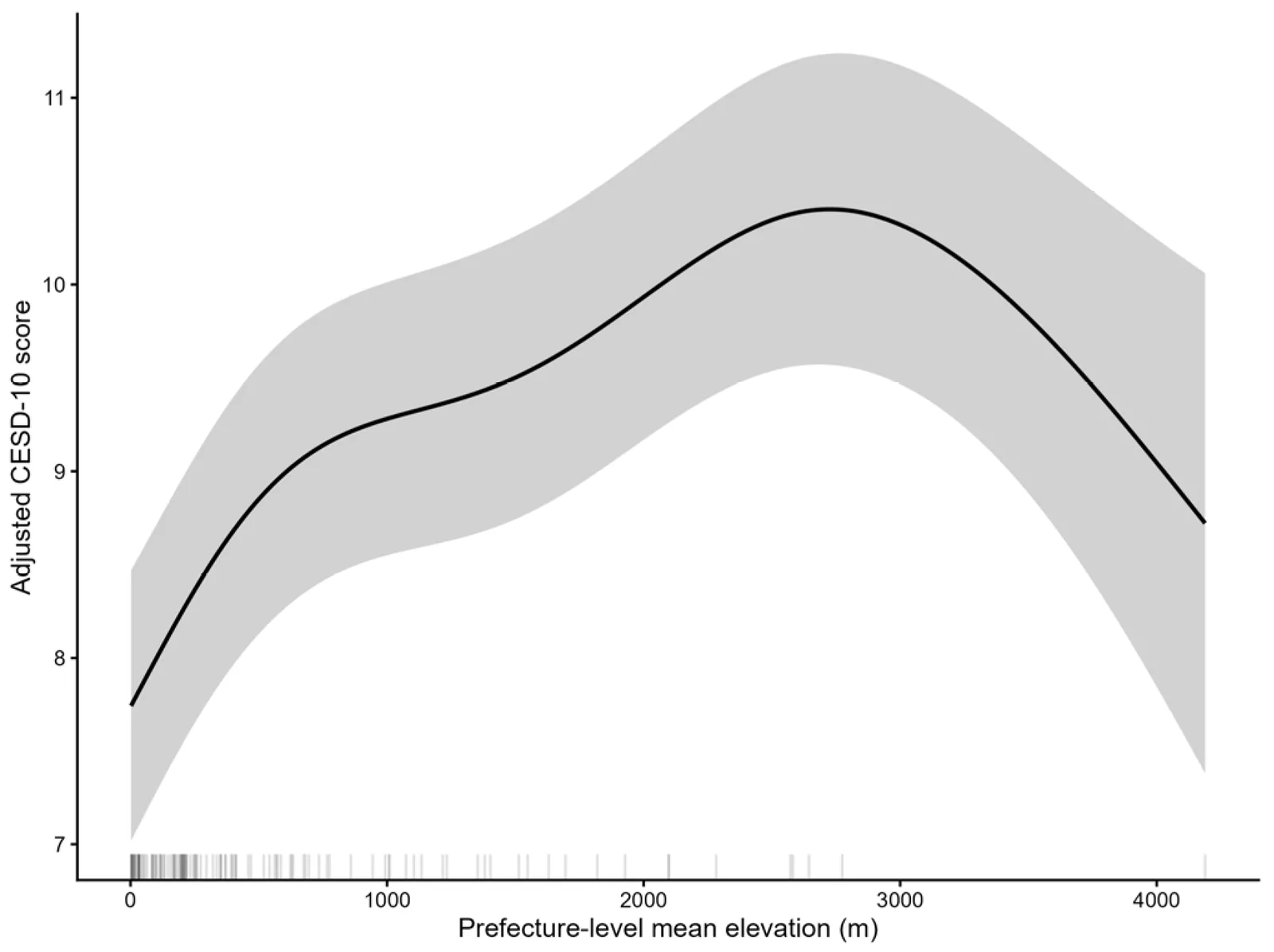
Non-linear dose-response associations between mean elevation and CESD-10 score. Panel A shows the GAM and Panel B the restricted cubic spline; shaded areas are 95% confidence intervals. Absolute predictions should not be compared across panels.

The adjusted GAM showed a nonlinear association between continuous prefecture-level mean elevation and CESD-10 score (edf = 3.742, F = 54.787, *p* < 0.001; Figure 5). A formal comparison of linear and smooth specifications also supported nonlinearity (F = 16.557, *p* < 0.001). Predicted CESD-10 scores increased across the lower-to-moderate elevation range and then leveled or declined at higher elevations. Confidence intervals widened substantially at the upper end of the elevation distribution, and these tail estimates should therefore be interpreted cautiously.

### 3.7. Exploratory Mediation Analysis of Sleep Duration

Exploratory mediation results are reported in Table S2 and Figures S6 and S7.

The estimated ACME was −0.115 (95% CI −0.225 to −0.008; *p* = 0.032) for moderate versus low elevation and −0.548 (95% CI −0.783 to −0.339; *p* < 0.001) for high versus low elevation. The corresponding ADEs and total effects were not statistically significant, and the estimated proportions mediated were highly imprecise (Table S2). Because the exposure, mediator, and outcome were assessed cross-sectionally, these findings should be regarded as exploratory statistical indirect effects rather than evidence of a causal or temporally ordered mediation pathway.

### 3.8. Exploratory Analyses Across Participant Characteristics

Most subgroup estimates by sex, age, and education were nonsignificant, and confidence intervals overlapped substantially (Table S3; Figure S5). No consistent subgroup pattern was evident. Without formal interaction tests, apparent differences between subgroups should be interpreted descriptively.

### 3.9. Robustness of the Main Findings in Sensitivity Analyses

Results were similar when altitude was reclassified using 1500 m, when altitude-by-exercise interactions were added, and when participants from Chaohu and Xiangfan were excluded (Table S4). None of these analyses produced a stable significant altitude main effect.

## 4. Discussion

In this large CHARLS sample, prefecture-level mean elevation was associated with higher CESD-10 scores in crude and conventional individual-level adjusted models, but these associations were substantially attenuated after accounting for province-level context. These associations were substantially attenuated and became statistically nonsignificant after sleep category and province fixed effects were added. Higher altitude was associated with slightly longer self-reported sleep duration, but the magnitude was small. Continuous sleep duration did not show a significant indirect effect, whereas the overall altitude-by-sleep interaction was statistically significant. Short sleepers had the highest adjusted CESD-10 predictions across all altitude groups. Continuous analyses also displayed curved relationships for both sleep duration and altitude, although the shape of the altitude curve was uncertain at the sparsely represented upper range.

Taken together, the findings do not support a simple model in which higher prefecture-level mean elevation independently increases depressive symptoms through a linear change in sleep duration. A more plausible interpretation is that geographic context and individual sleep patterns jointly contribute to the observed distribution of depressive symptoms, while prefecture-level mean elevation may partly capture broader regional differences. This distinction is important because a statistically visible geographic gradient in a crude model does not necessarily identify the environmental component responsible for that gradient.

Previous studies have reported associations between altitude and depressive symptoms, but differences in exposure definition, population characteristics, regional context, and adjustment strategies make direct comparison difficult [11,12,17,18,19,20]. Evidence from ecological studies has also linked altitude with suicide-related outcomes, but these findings may be influenced by regional socioeconomic differences [13,15,16].

The attenuation of the elevation coefficient after regional adjustment does not indicate that geographic context is irrelevant to mental health. Rather, it indicates that the contrast represented by prefecture-level altitude was strongly entangled with sleep category and province-level background. Elevation in China is spatially patterned and may co-vary with health-care access, educational opportunities, labor and migration patterns, urbanization, transportation, climate, and social support. Province fixed effects control characteristics shared within provinces, including many stable policy, cultural, and development differences, while leaving contrasts among prefectures within the same province. The attenuation observed here therefore suggests that part of the association observed in previous models may reflect broader regional contrasts rather than altitude itself [21]. At the same time, fixed effects are not a complete solution to spatial confounding. Prefecture-level conditions that vary within provinces may remain unmeasured, and province adjustment may also reduce the precision of altitude estimates when within-province elevation variation is limited. The finding is consequently best described as a lack of a robust independent altitude association under the specified model rather than proof of no environmental contribution.

Acute altitude studies have consistently reported impaired sleep continuity, periodic breathing, and nocturnal oxygen desaturation [5,6,9,10]. Our finding of slightly longer self-reported sleep at moderate and high altitude may appear inconsistent with that literature, but the outcomes are not equivalent. Sleep duration does not measure sleep quality, fragmentation, respiratory disturbance, or restorative value. A person may spend more time sleeping or in bed while experiencing poorer sleep efficiency. In addition, long-term residents differ fundamentally from recently exposed lowlanders because of physiological acclimatization, established daily routines, housing conditions, occupation, and culturally patterned sleep schedules.

The adjusted differences of 0.280 and 0.363 h correspond to approximately 17 and 22 min per day. Although statistically detectable in a large sample, these effect sizes are modest and should not be interpreted as evidence that altitude improves sleep. They may reflect behavioral compensation for poorer sleep quality, differences in work and daylight schedules, or residual regional confounding. Background/Objectives measures such as actigraphy, polysomnography, nocturnal oxygen saturation, sleep efficiency, and sleep-disordered breathing would be required to distinguish these possibilities.

Sleep duration was strongly associated with depressive symptoms, consistent with prospective and meta-analytic evidence linking insufficient sleep and insomnia to poorer mental health [1,2,3,4]. Nevertheless, the exploratory mediation analysis did not provide evidence for a stable linear indirect pathway from prefecture-level mean elevation through continuous sleep duration to CESD-10 score. Several explanations are possible. First, altitude may affect sleep quality or nocturnal oxygenation without materially changing total duration. Second, a single self-reported duration measure may contain substantial measurement error. Third, sleep and depressive symptoms were assessed cross-sectionally, so reverse causation and temporal ambiguity are unavoidable. Finally, although province fixed effects were incorporated into the revised mediation models, the cross-sectional design limits causal interpretation, and the exploratory mediation estimates should not be interpreted as evidence of a temporal or biological pathway.

The significant overall interaction addresses a different question. It indicates that the adjusted pattern across altitude groups was not identical in short, normal, and long sleepers. Short sleepers consistently had the highest predicted CESD-10 scores, suggesting that insufficient sleep identifies a subgroup with elevated depressive symptom burden across geographic settings. The lower predictions among long sleepers at moderate and high altitude should not be interpreted as a causal protective effect. Long sleep can reflect heterogeneous states, including recovery, low activity, chronic illness, or reverse causation, and the relatively small number of long sleepers at high altitude increases uncertainty. The GAM result, which showed higher predicted scores at both very short and very long durations, further argues against a simple “more sleep is better” interpretation.

From an etiologic perspective, sleep may function as a marker of vulnerability rather than as the mechanism transmitting an altitude effect. Sleep restriction can impair emotion regulation, increase stress reactivity, and reduce coping capacity, which could magnify the mental-health consequences of adverse social or environmental conditions. This interpretation remains provisional because statistical interaction on the additive score scale does not establish biological interaction.

The continuous analyses were included to reduce dependence on prespecified altitude categories. Both models showed greater CESD-10 predictions across part of the low-to-moderate elevation range, followed by flattening, but the GAM and restricted cubic spline differed at the upper end. Such differences can arise from alternative smoothing assumptions, knot placement, sparse observations, and extrapolation near the boundaries. Formal comparisons supported nonlinearity for both sleep duration and prefecture-level mean elevation; however, confidence intervals widened substantially at high altitude, and differences between GAM and restricted cubic spline estimates suggest uncertainty related to smoothing assumptions and sparse observations. Therefore, the curves should be viewed as descriptive rather than as evidence of a specific biological threshold.

The main >2000 m category and the ≥1500 m sensitivity threshold represent pragmatic epidemiological contrasts rather than universal clinical cut points [27,28,29]. The consistency of the null fully adjusted main effect under the alternative classification reduces concern that the conclusion was created solely by the selected cutoff. Nevertheless, prefecture-level mean elevation smooths substantial within-area variation, so an apparent plateau cannot be interpreted as an individual physiological adaptation threshold.

The study has two practical implications. First, geographic differences in depressive symptoms should not automatically be attributed to a single natural exposure. Environmental public-health analyses should jointly consider physical geography, regional resources, health-care accessibility, and social conditions. For higher-altitude regions, improving mental-health services may depend as much on access, screening capacity, and social support as on altitude-related physiology.

Second, short sleep was associated with a high predicted depressive symptom score at every altitude. Sleep duration is not a substitute for clinical assessment, but it is a simple characteristic that can be incorporated into community and primary-care screening. Middle-aged and older adults reporting persistently short sleep may warrant assessment for depressive symptoms, insomnia, sleep-disordered breathing, chronic disease burden, and social stressors. The present data do not demonstrate that extending sleep would reduce depression, but they support considering sleep health within geographically tailored mental-health strategies.

Strengths of this study include the large and geographically diverse CHARLS sample, use of a fixed analytical cohort across models, linkage of individual survey data with a spatial environmental measure, and a structured analysis that compared conventional adjustment with province fixed effects. Interaction, mediation, nonlinear, subgroup, and sensitivity analyses addressed complementary questions rather than relying on a single model. The explicit distinction between model-based predictions and raw group means also reduces the risk of misinterpreting the interaction results.

Several limitations require emphasis. First, the cross-sectional design prevents determination of temporal order and precludes causal interpretation, particularly for mediation and the direction of the sleep-depression association. Second, sleep duration was self-reported and did not capture sleep quality, insomnia symptoms, apnea, nocturnal hypoxia, sleep efficiency, daytime napping, or sleep architecture. Third, CESD-10 measures depressive symptoms rather than a clinical diagnosis. Fourth, prefecture-level mean elevation is an area-level proxy and may misclassify the altitude of individual homes, workplaces, and daily activity locations [21,25]. Participants were also nested within prefectures; future analyses should verify uncertainty estimates using prefecture-clustered or multilevel approaches.

Fifth, complete-case selection may have introduced selection bias, and unmeasured factors such as income, occupation, ethnicity, migration history, medication use, detailed health-care access, temperature, air pollution, and social support may still confound the results. Province fixed effects control stable province-level characteristics but not all time-varying or prefecture-specific conditions. Sixth, sleep category and province fixed effects were introduced together in Model 4, so their separate contributions to coefficient attenuation could not be quantified. Seventh, the high-altitude and long-sleep subgroups were relatively small, subgroup contrasts were not supported by formal interaction tests, and no multiplicity correction was applied to the secondary exploratory analyses. Finally, the nonlinear curves were model-dependent and imprecise at the exposure extremes. These limitations reinforce the need for longitudinal studies using individual geocoded altitude, objective multidimensional sleep assessment, and more detailed contextual measures [41,42,43].

## 5. Conclusions

In this cross-sectional CHARLS study, higher prefecture-level mean elevation was associated with greater depressive symptom scores in crude and conventional covariate-adjusted models, but this association was substantially attenuated after adjustment for province-level context and sleep category. These findings do not support interpreting prefecture-level mean elevation as an independent determinant of depressive symptoms. Higher prefecture-level mean elevation was associated with slightly longer self-reported sleep, but continuous sleep duration did not show evidence of mediation. The elevation-by-sleep interaction suggests that sleep category modifies the observed pattern of depressive symptoms across geographic contexts, with short sleepers showing the highest adjusted scores across elevation groups. Longitudinal research combining individual-level elevation, objective sleep measures, regional social and environmental data, and appropriately clustered or multilevel analyses is needed to distinguish physiological, contextual, and behavioral pathways. From a public-health perspective, regional mental-health planning should address both local structural conditions and sleep-related vulnerability.

## Figures and Tables

**Table 1:** Baseline characteristics according to prefecture-level mean elevation categories.

	Overall N = 15,562 ^1^	Low (<1000 m) N = 12,453 ^1^	Moderate (1000–2000 m) N = 2112 ^1^	High (>2000 m) N = 997 ^1^	*p*-Value ^2^
Age, years	61.7 (9.5)	62.1 (9.5)	60.9 (9.6)	59.8 (9.2)	<0.001
Sleep duration, hours	6.3 (1.9)	6.2 (1.8)	6.3 (2.0)	6.3 (2.0)	0.065
CESD-10 score	8.2 (6.4)	7.9 (6.2)	9.3 (6.7)	9.9 (6.6)	<0.001
Sex					0.742
Female	7846 (50%)	6283 (50%)	1052 (50%)	511 (51%)	
Male	7716 (50%)	6170 (50%)	1060 (50%)	486 (49%)	
Education level					<0.001
Low (≤Primary)	10,665 (69%)	8358 (67%)	1541 (73%)	766 (77%)	
Middle or higher (≥Junior)	4897 (31%)	4095 (33%)	571 (27%)	231 (23%)	
Smoking	6813 (44%)	5400 (43%)	1004 (48%)	409 (41%)	<0.001
Alcohol drinking	7686 (49%)	6252 (50%)	939 (44%)	495 (50%)	<0.001
Chronic disease	12,354 (79%)	9849 (79%)	1712 (81%)	793 (80%)	0.116
Pension	9451 (61%)	7771 (62%)	1196 (57%)	484 (49%)	<0.001
Medical insurance	15,184 (98%)	12,153 (98%)	2061 (98%)	970 (97%)	0.839
Residence					<0.001
Rural	9402 (60%)	7381 (59%)	1368 (65%)	653 (65%)	
Urban	6160 (40%)	5072 (41%)	744 (35%)	344 (35%)	
Regular exercise	14,331 (92%)	11,424 (92%)	1965 (93%)	942 (94%)	0.002
Marital status					0.100
Married	13,490 (87%)	10,829 (87%)	1801 (85%)	860 (86%)	
Unmarried	2072 (13%)	1624 (13%)	311 (15%)	137 (14%)	
Sleep duration category					<0.001
Short (<6 h)	5247 (34%)	4196 (34%)	730 (35%)	321 (32%)	
Normal (6–8 h)	8927 (57%)	7205 (58%)	1160 (55%)	562 (56%)	
Long (>8 h)	1388 (8.9%)	1052 (8.4%)	222 (11%)	114 (11%)	

^1^ Mean (SD); n (%); ^2^ Kruskal-Wallis rank sum test; Pearson’s Chi-squared test. Values are presented as mean (SD) or n (%). *p* values were calculated using Kruskal–Wallis tests for continuous variables and Pearson’s chi-squared tests for categorical variables.

**Table 2:** Associations between prefecture-level mean elevation categories and CESD-10 scores across sequential adjustment models.

Model	Moderate Elevation, β (95% CI)	*p* (Moderate)	High Elevation, β (95% CI)	*p* (High)
Model 1	1.409 (0.376, 2.443)	0.008	1.997 (0.859, 3.136)	<0.001
Model 2	1.376 (0.369, 2.383)	0.008	1.917 (0.790, 3.043)	0.001
Model 3	1.176 (0.295, 2.058)	0.009	1.771 (0.808, 2.733)	<0.001
Model 4a	−0.368 (−1.516, 0.780)	0.527	−0.369 (−1.670, 0.932)	0.575
Model 4b	1.153 (0.339, 1.968)	0.006	1.811 (0.886, 2.735)	<0.001
Model 4c	−0.288 (−1.299, 0.723)	0.574	−0.174 (−1.254, 0.906)	0.751

Values are β coefficients (95% CIs); low elevation (<1000 m) is the reference. All standard errors are clustered at the prefecture level. Model 1: unadjusted. Model 2: adjusted for age, sex, and education. Model 3: further adjusted for smoking, alcohol drinking, chronic disease, pension, medical insurance, residence, regular exercise, and marital status. Model 4a: Model 3 + province fixed effects. Model 4b: Model 3 + sleep-duration category. Model 4c: Model 3 + sleep-duration category + province fixed effects.

**Table 3:** Interaction between prefecture-level mean elevation and sleep duration categories on CESD-10 scores.

Variable	β (95% CI)	*p*
Moderate elevation × Normal sleep	−0.604 (−1.340, 0.132)	0.107
High elevation × Normal sleep	−0.336 (−1.331, 0.659)	0.505
Moderate elevation × Long sleep	−1.549 (−2.671, −0.427)	0.007
High elevation × Long sleep	−1.908 (−3.444, −0.372)	0.015

Model adjusted for prespecified individual-level covariates and province fixed effects; standard errors were clustered at the prefecture level. Joint Wald test: χ^2^ = 17.022, df = 4, *p* = 0.002.

## Data Availability

The data analyzed in this study were obtained from the China Health and Retirement Longitudinal Study (CHARLS). CHARLS datasets are available to researchers upon application through the official CHARLS website (https://charls.charlsdata.com). The datasets used in this study can be accessed after registration and approval according to the CHARLS data access policy.

## Supplementary Materials

The following supporting information can be downloaded at: https://www.mdpi.com/article/10.3390/healthcare14203544/s1, Supplementary Figures S1–S9 and Supplementary Tables S1–S9. Table S1. Association between elevation and total daily sleep duration; Table S2. Full coefficients from the primary regression models; Table S3. Full elevation × sleep-duration interaction model; Table S4. Adjusted predicted CESD-10 scores by elevation and sleep-duration category; Table S5. Formal tests for nonlinearity; Table S6. Generalized additive model smooth-term results; Table S7. Exploratory subgroup analyses; Table S8. Sensitivity analyses; Table S9. Exploratory mediation analysis with province fixed effects and 2000 bootstrap replications; Figure S1. Directed acyclic graph of the hypothesized relationships among area-level altitude, sleep duration, depressive symptoms, and covariates. Gray nodes indicate covariates; green, purple, and orange nodes represent altitude, sleep, and depressive symptoms, respectively. Figure S2. Distribution of self-reported sleep duration by altitude group. The Kruskal–Wallis test indicated no statistically significant overall difference in continuous sleep duration among the three groups (P = 0.065). Figure S3. Marginal-standardized predicted CESD-10 scores for combinations of altitude group and sleep category. Predictions were obtained from the fully adjusted altitude × sleep interaction model. Figure S4. Fully adjusted association between altitude group and CESD-10 score. Points represent β coefficients and horizontal lines represent 95% confidence intervals; low altitude was the reference group. Figure S5. Subgroup-specific associations between altitude group and CESD-10 score. Estimates are exploratory; no formal between-subgroup interaction tests were reported. Figure S6. Exploratory mediation analysis comparing moderate with low altitude. ACME denotes the indirect effect through continuous sleep duration and ADE denotes the direct effect. Figure S7. Exploratory mediation analysis comparing high with low altitude. ACME denotes the indirect effect through continuous sleep duration and ADE denotes the direct effect. Figure S8. Sensitivity Analyses Assessing the Robustness of the Primary Findings. Figure S9. Subgroup, mediation, nonlinear, and sensitivity analyses were considered exploratory and were interpreted cautiously.
