# Supplementary material for: Prefecture-Level Mean Elevation, Sleep Duration, and Depressive Symptoms Among Middle-Aged and Older Adults in China: A Cross-Sectional CHARLS Study
Source: Healthcare (Basel). 2026 Oct 21;14(20):3544. doi: 10.3390/healthcare14203544 (PMC13704447; doi:10.3390/healthcare14203544)
Supplement: Supplementary file 1 [file healthcare-14-03544-s001.zip › healthcare-4550098-supplementary.pdf]

Supplementary Materials

Supplementary analytical notes

All primary linear regression and elevation-by-sleep interaction models used prefecture-clustered robust standard errors. Province fixed effects were included where indicated. Model 1 was unadjusted; Model 2 adjusted for age, sex, and education; Model 3 further adjusted for smoking, alcohol drinking, chronic disease, pension, medical insurance, residence, regular exercise, and marital status; Model 4a added province fixed effects to Model 3; Model 4b added sleep-duration category to Model 3; and Model 4c included both sleep-duration category and province fixed effects. Low elevation (<1,000 m), short sleep (<6 h), female sex, low education, and the corresponding absence/reference categories for binary covariates were the reference groups. Subgroup, mediation, nonlinear, and sensitivity analyses were exploratory.

Table S1. Association between elevation and total daily sleep duration

Exposure	Difference in sleep duration, h (95% CI)	P
Moderate elevation (1,000–2,000 m)	0.280 (-0.012, 0.571)	0.060
High elevation (>2,000 m)	0.363 (-0.109, 0.834)	0.130

Low elevation (<1,000 m) was the reference group. Models adjusted for prespecified individual-level covariates and province fixed effects. Standard errors were clustered at the prefecture level. Sleep duration represents total daily sleep including naps.

Table S2. Full coefficients from the primary regression models

Model	Variable	β (95% CI)	P
Model 1	(Intercept)	7.901 (7.577, 8.226)	<0.001
Model 1	Moderate elevation (1,000–2,000 m)	1.409 (0.376, 2.443)	0.008
Model 1	High elevation (>2,000 m)	1.997 (0.859, 3.136)	<0.001
Model 2	(Intercept)	7.278 (6.275, 8.281)	<0.001
Model 2	Moderate elevation (1,000–2,000 m)	1.376 (0.369, 2.383)	0.008
Model 2	High elevation (>2,000 m)	1.917 (0.790, 3.043)	0.001
Model 2	age	0.033 (0.018, 0.047)	<0.001
Model 2	Male	-1.899 (-2.097, -1.701)	<0.001
Model 2	Middle or higher education	-1.445 (-1.736, -1.154)	<0.001
Model 3	(Intercept)	8.850 (7.710, 9.990)	<0.001
Model 3	Moderate elevation (1,000–2,000 m)	1.176 (0.295, 2.058)	0.009
Model 3	High elevation (>2,000 m)	1.771 (0.808, 2.733)	<0.001
Model 3	age	0.006 (-0.010, 0.021)	0.459
Model 3	Male	-2.205 (-2.484, -1.925)	<0.001
Model 3	Middle or higher education	-1.028 (-1.279, -0.777)	<0.001
Model 3	Smoking	0.611 (0.332, 0.890)	<0.001
Model 3	Alcohol drinking	-0.144 (-0.413, 0.124)	0.290
Model 3	Chronic disease	2.397 (2.120, 2.674)	<0.001
Model 3	Pension	-0.283 (-0.607, 0.040)	0.085
Model 3	Medical insurance	-0.585 (-1.282, 0.111)	0.099
Model 3	Urban residence	-1.562 (-1.896, -1.229)	<0.001
Model 3	Regular exercise	-0.806 (-1.177, -0.435)	<0.001
Model 3	Unmarried	1.246 (0.869, 1.623)	<0.001
Model 4a	Moderate elevation (1,000–2,000 m)	-0.368 (-1.516, 0.780)	0.527
Model 4a	High elevation (>2,000 m)	-0.369 (-1.670, 0.932)	0.575
Model 4a	age	0.002 (-0.012, 0.017)	0.753
Model 4a	Male	-2.170 (-2.450, -1.889)	<0.001
Model 4a	Middle or higher education	-0.984 (-1.228, -0.739)	<0.001
Model 4a	Smoking	0.599 (0.327, 0.870)	<0.001
Model 4a	Alcohol drinking	-0.187 (-0.441, 0.067)	0.147
Model 4a	Chronic disease	2.294 (2.039, 2.549)	<0.001
Model 4a	Pension	-0.239 (-0.548, 0.069)	0.127
Model 4a	Medical insurance	-0.566 (-1.226, 0.093)	0.092
Model 4a	Urban residence	-1.486 (-1.779, -1.192)	<0.001

Model 4a	Regular exercise	-0.952 (-1.319, -0.585)	<0.001
Model 4a	Unmarried	1.228 (0.854, 1.603)	<0.001
Model 4b	(Intercept)	11.470 (10.349, 12.590)	<0.001
Model 4b	Moderate elevation (1,000–2,000 m)	1.153 (0.339, 1.968)	0.006
Model 4b	High elevation (>2,000 m)	1.811 (0.886, 2.735)	<0.001
Model 4b	Normal sleep (6–8 h)	-2.957 (-3.202, -2.712)	<0.001
Model 4b	Long sleep (>8 h)	-3.156 (-3.538, -2.774)	<0.001
Model 4b	age	-0.004 (-0.018, 0.010)	0.586
Model 4b	Male	-1.842 (-2.135, -1.550)	<0.001
Model 4b	Middle or higher education	-0.953 (-1.190, -0.717)	<0.001
Model 4b	Smoking	0.548 (0.287, 0.809)	<0.001
Model 4b	Alcohol drinking	-0.221 (-0.487, 0.045)	0.102
Model 4b	Chronic disease	2.110 (1.846, 2.373)	<0.001
Model 4b	Pension	-0.281 (-0.584, 0.021)	0.068
Model 4b	Medical insurance	-0.574 (-1.204, 0.057)	0.074
Model 4b	Urban residence	-1.530 (-1.846, -1.215)	<0.001
Model 4b	Regular exercise	-0.756 (-1.119, -0.393)	<0.001
Model 4b	Unmarried	1.063 (0.695, 1.432)	<0.001
Model 4c	Moderate elevation (1,000–2,000 m)	-0.288 (-1.299, 0.723)	0.574
Model 4c	High elevation (>2,000 m)	-0.174 (-1.254, 0.906)	0.751
Model 4c	Normal sleep (6–8 h)	-2.875 (-3.114, -2.636)	<0.001
Model 4c	Long sleep (>8 h)	-3.084 (-3.467, -2.701)	<0.001
Model 4c	age	-0.006 (-0.020, 0.008)	0.381
Model 4c	Male	-1.811 (-2.100, -1.522)	<0.001
Model 4c	Middle or higher education	-0.936 (-1.168, -0.704)	<0.001
Model 4c	Smoking	0.520 (0.265, 0.776)	<0.001
Model 4c	Alcohol drinking	-0.248 (-0.500, 0.004)	0.054
Model 4c	Chronic disease	2.037 (1.798, 2.277)	<0.001
Model 4c	Pension	-0.245 (-0.534, 0.044)	0.096
Model 4c	Medical insurance	-0.568 (-1.170, 0.034)	0.064
Model 4c	Urban residence	-1.458 (-1.745, -1.171)	<0.001
Model 4c	Regular exercise	-0.872 (-1.225, -0.519)	<0.001
Model 4c	Unmarried	1.048 (0.682, 1.414)	<0.001

All primary models use prefecture-clustered robust standard errors.

Note. Intercepts are omitted for compactness. All confidence intervals are based on prefecture-clustered robust covariance estimates. Model 4a = Model 3 + province fixed effects; Model 4b = Model 3 + sleep-duration category; Model 4c = Model 3 + sleep-duration category + province fixed effects.

Table S3. Full elevation × sleep-duration interaction model

Variable	β (95% CI)	P
Moderate elevation (1,000–2,000 m)	0.204 (-0.969, 1.377)	0.731
High elevation (>2,000 m)	0.206 (-1.042, 1.455)	0.744
Normal sleep (6–8 h)	-2.772 (-3.034, -2.510)	<0.001
Long sleep (>8 h)	-2.696 (-3.060, -2.331)	<0.001
age	-0.007 (-0.021, 0.007)	0.343
Male	-1.803 (-2.090, -1.517)	<0.001
Middle or higher education	-0.932 (-1.164, -0.700)	<0.001
Smoking	0.516 (0.262, 0.770)	<0.001
Alcohol drinking	-0.249 (-0.501, 0.002)	0.052
Chronic disease	2.038 (1.798, 2.279)	<0.001
Pension	-0.243 (-0.532, 0.046)	0.098
Medical insurance	-0.549 (-1.142, 0.043)	0.069
Urban residence	-1.454 (-1.742, -1.166)	<0.001
Regular exercise	-0.882 (-1.232, -0.531)	<0.001
Unmarried	1.048 (0.683, 1.413)	<0.001

altitude_groupModerate (1,000–2,000 m):sleep_catNormal (6–8 h)	-0.604 (-1.340, 0.132)	0.107
altitude_groupHigh (>2,000 m):sleep_catNormal (6–8 h)	-0.336 (-1.331, 0.659)	0.505
altitude_groupModerate (1,000–2,000 m):sleep_catLong (>8 h)	-1.549 (-2.671, -0.427)	0.007
altitude_groupHigh (>2,000 m):sleep_catLong (>8 h)	-1.908 (-3.444, -0.372)	0.015

Province fixed effects included; standard errors clustered at the prefecture level.

Note. Province fixed effects were included, and standard errors were clustered at the prefecture level. Reference group: low elevation (<1,000 m) and short sleep (<6 h). Overall interaction: joint Wald χ^2 = 17.022, df = 4, P = 0.002.

Table S4. Adjusted predicted CESD-10 scores by elevation and sleep-duration category

Elevation group	Sleep category	Predicted CESD-10 (95% CI)
Low (<1,000 m)	Short (<6 h)	10.10 (9.81, 10.40)
Low (<1,000 m)	Normal (6–8 h)	7.33 (7.07, 7.59)
Low (<1,000 m)	Long (>8 h)	7.41 (7.04, 7.77)
Moderate (1,000–2,000 m)	Short (<6 h)	10.31 (9.24, 11.38)
Moderate (1,000–2,000 m)	Normal (6–8 h)	6.93 (6.10, 7.76)
Moderate (1,000–2,000 m)	Long (>8 h)	6.06 (4.77, 7.35)
High (>2,000 m)	Short (<6 h)	10.31 (9.21, 11.41)
High (>2,000 m)	Normal (6–8 h)	7.20 (6.26, 8.15)
High (>2,000 m)	Long (>8 h)	5.71 (4.42, 6.99)

Adjusted estimated marginal means from a model including province indicators. Confidence intervals use prefecture-clustered robust covariance estimates.

Table S5. Formal tests for nonlinearity

Exposure	Df_change	F	P
Total daily sleep duration	3.059	89.756	<0.001
Prefecture-level mean elevation	3.182	16.557	<0.001

Formal nonlinearity was evaluated by comparing linear and smooth GAM specifications fitted by maximum likelihood using an F test.

Table S6. Generalized additive model smooth-term results

Exposure	edf	Ref_df	F	P
Total daily sleep duration	3.848	3.986	296.762	<0.001
Prefecture-level mean elevation	3.742	3.961	54.787	<0.001

Final GAMs used penalized smooths with k=5 and REML estimation.

Table S7. Exploratory subgroup analyses

Subgroup	N	Elevation	β (95% CI)	P
Female	7,846	Moderate (1,000–2,000 m)	-0.687 (-1.861, 0.488)	0.249
Female	7,846	High (>2,000 m)	-0.035 (-1.715, 1.645)	0.967
Male	7,716	Moderate (1,000–2,000 m)	0.102 (-0.882, 1.085)	0.838
Male	7,716	High (>2,000 m)	-0.298 (-1.391, 0.794)	0.590
Age <60	6,604	Moderate (1,000–2,000 m)	-0.701 (-1.831, 0.430)	0.222
Age <60	6,604	High (>2,000 m)	-0.495 (-1.667, 0.677)	0.405
Age ≥60	8,958	Moderate (1,000–2,000 m)	0.251 (-0.871, 1.372)	0.659
Age ≥60	8,958	High (>2,000 m)	0.422 (-0.918, 1.763)	0.534
Low education	10,665	Moderate (1,000–2,000 m)	-0.144 (-1.322, 1.033)	0.809
Low education	10,665	High (>2,000 m)	0.099 (-1.116, 1.314)	0.872
Middle+ education	4,897	Moderate (1,000–2,000 m)	-0.712 (-1.982, 0.557)	0.269
Middle+ education	4,897	High (>2,000 m)	-1.322 (-3.366, 0.722)	0.203

Subgroup analyses are exploratory. Province fixed effects were included and standard errors were clustered at the prefecture level. No formal between-subgroup interaction tests or multiple-comparison correction were applied.

Table S8. Sensitivity analyses

Analysis	Variable	β (95% CI)	P
Alternative 1,500-m threshold	Middle elevation (1,000–1,500 m)	-0.260 (-1.420, 0.900)	0.658
Alternative 1,500-m threshold	High elevation (≥1,500 m)	-0.261 (-1.289, 0.767)	0.616
Elevation × exercise interaction	Moderate elevation (1,000–2,000 m)	0.014 (-1.484, 1.512)	0.985
Elevation × exercise interaction	High elevation (>2,000 m)	-0.405 (-2.739, 1.928)	0.732
Elevation × exercise interaction	Moderate elevation (1,000–2,000 m):exercise	-0.321 (-1.757, 1.115)	0.659
Elevation × exercise interaction	High elevation (>2,000 m):exercise	0.246 (-1.745, 2.237)	0.807

Excluding Chaohu and Xiangfan	Moderate elevation (1,000–2,000 m)	-0.288 (-1.299, 0.723)	0.574
Excluding Chaohu and Xiangfan	High elevation (>2,000 m)	-0.174 (-1.254, 0.906)	0.751

All sensitivity models included province fixed effects where applicable and used prefecture-clustered robust standard errors.

Table S9. Exploratory mediation analysis with province fixed effects and 2,000 bootstrap replications

Elevation comparison	Indirect effect (95% CI)	P	Direct/total effects	Proportion mediated
Moderate vs low elevation	-0.115 (-0.225, -0.008)	0.032	Not statistically significant	Highly imprecise
High vs low elevation	-0.548 (-0.783, -0.339)	<0.001	Not statistically significant	Highly imprecise

Note. Continuous total daily sleep duration was used as the mediator. Province fixed effects were included in both the mediator and outcome models. Confidence intervals were obtained from 2,000 nonparametric bootstrap replications. Because the analysis is cross-sectional, these estimates describe exploratory statistical indirect effects and should not be interpreted as causal mediation. The direct and total effects were not statistically significant, and estimates of the proportion mediated were highly imprecise.

Supplementary Materials: Supplementary Figures

Residential altitude, sleep duration, and depressive symptoms among middle-aged and older adults in China

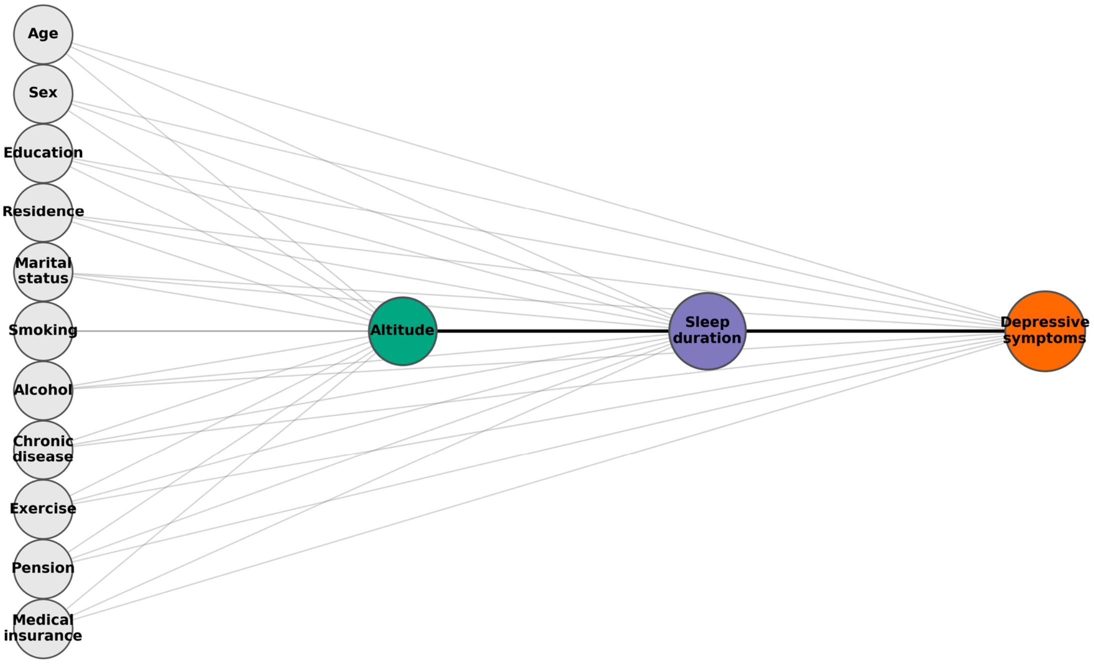

Figure S1. Directed acyclic graph of the hypothesized relationships among area-level altitude, sleep duration, depressive symptoms, and covariates. Gray nodes indicate covariates; green, purple, and orange nodes represent altitude, sleep, and depressive symptoms, respectively.

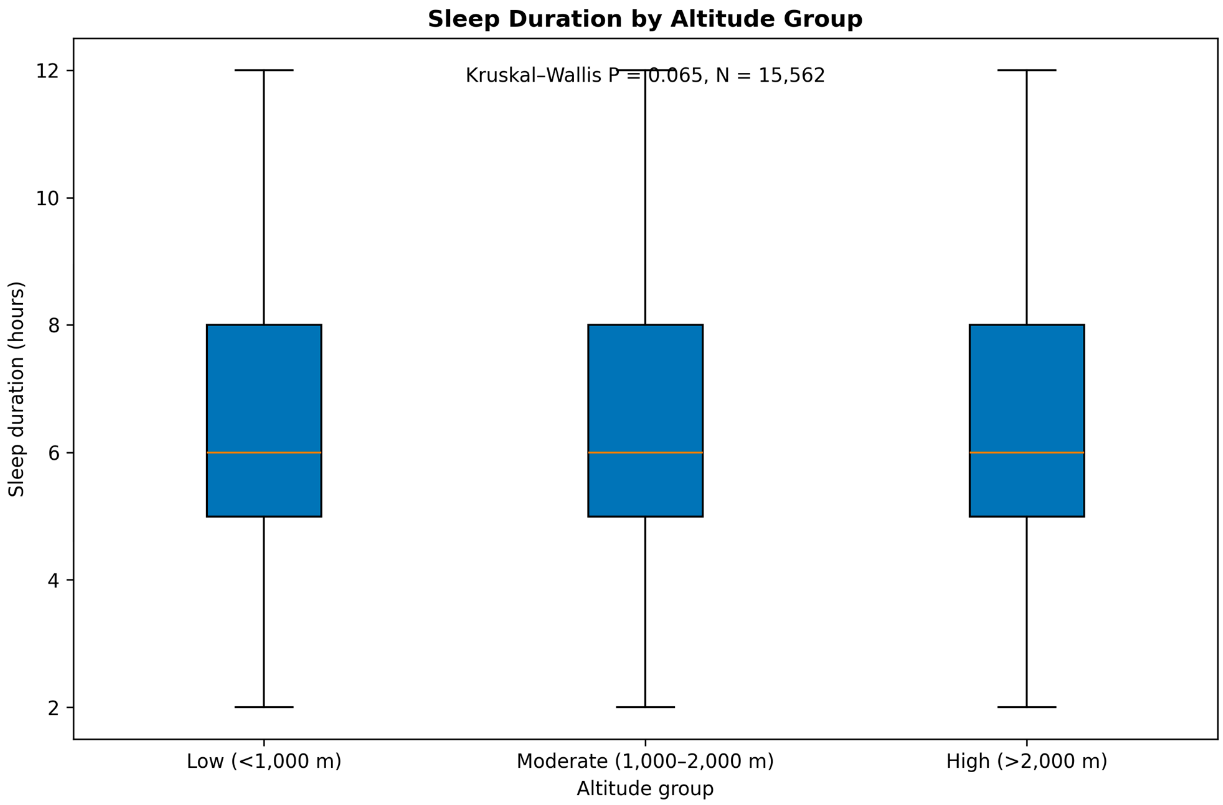

Figure S2. Distribution of self-reported sleep duration by altitude group. The Kruskal–Wallis test indicated no statistically significant overall difference in continuous sleep duration among the three groups (P = 0.065).

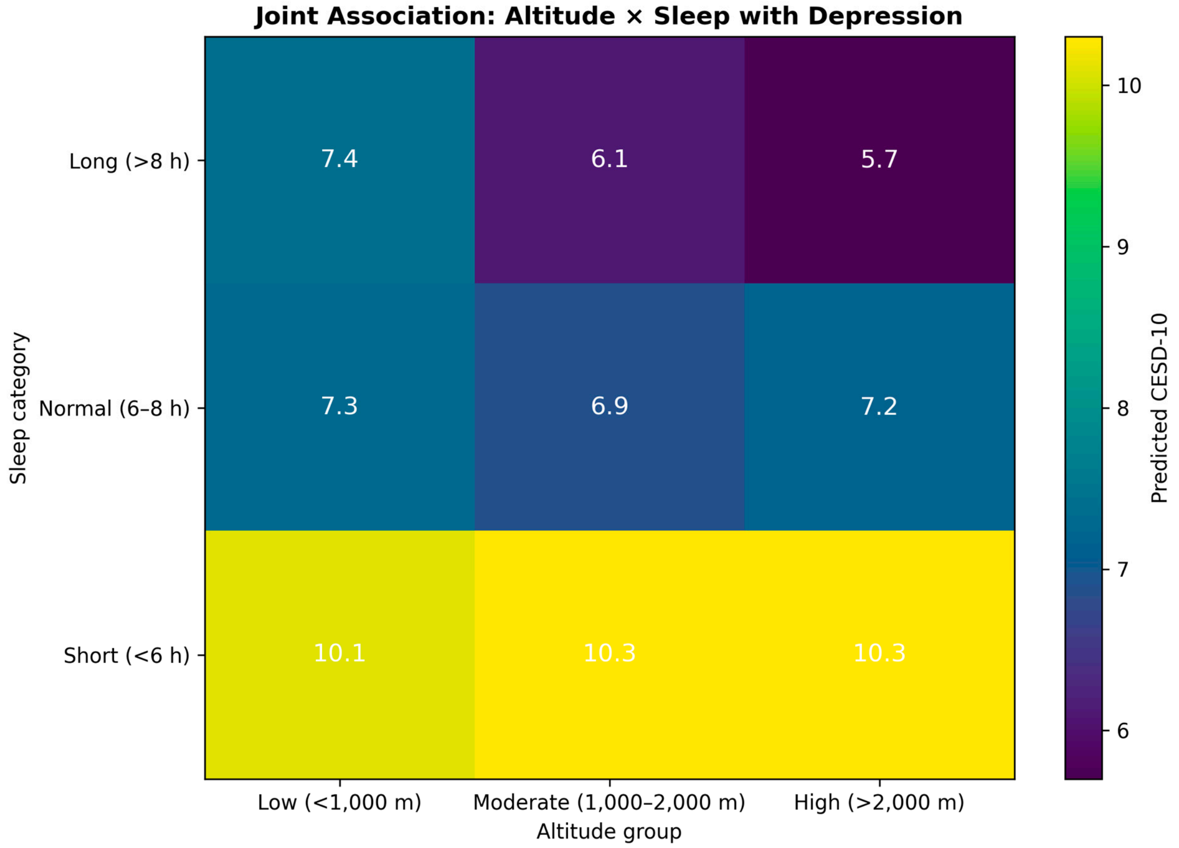

Figure S3. Marginal-standardized predicted CESD-10 scores for combinations of altitude group and sleep category. Predictions were obtained from the fully adjusted altitude × sleep interaction model.

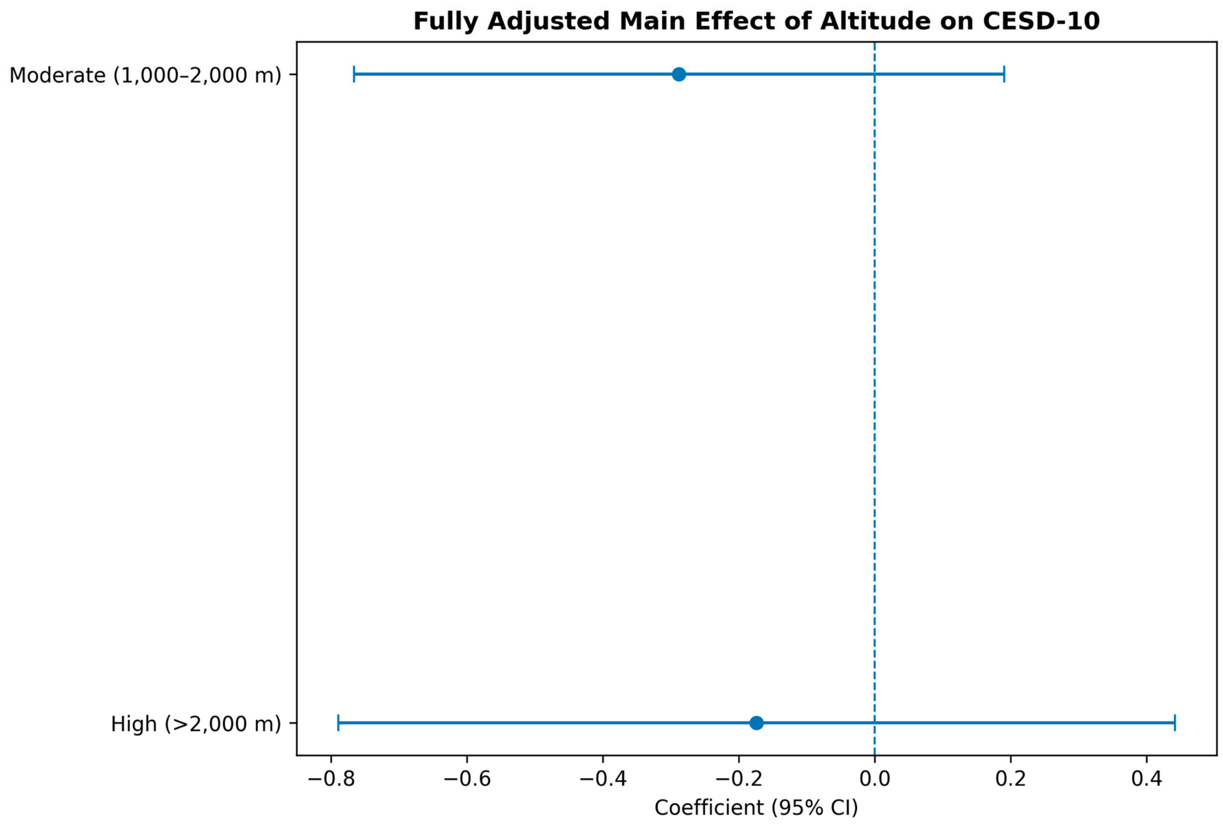

Figure S4. Fully adjusted association between altitude group and CESD-10 score. Points represent β coefficients and horizontal lines represent 95% confidence intervals; low altitude was the reference group.

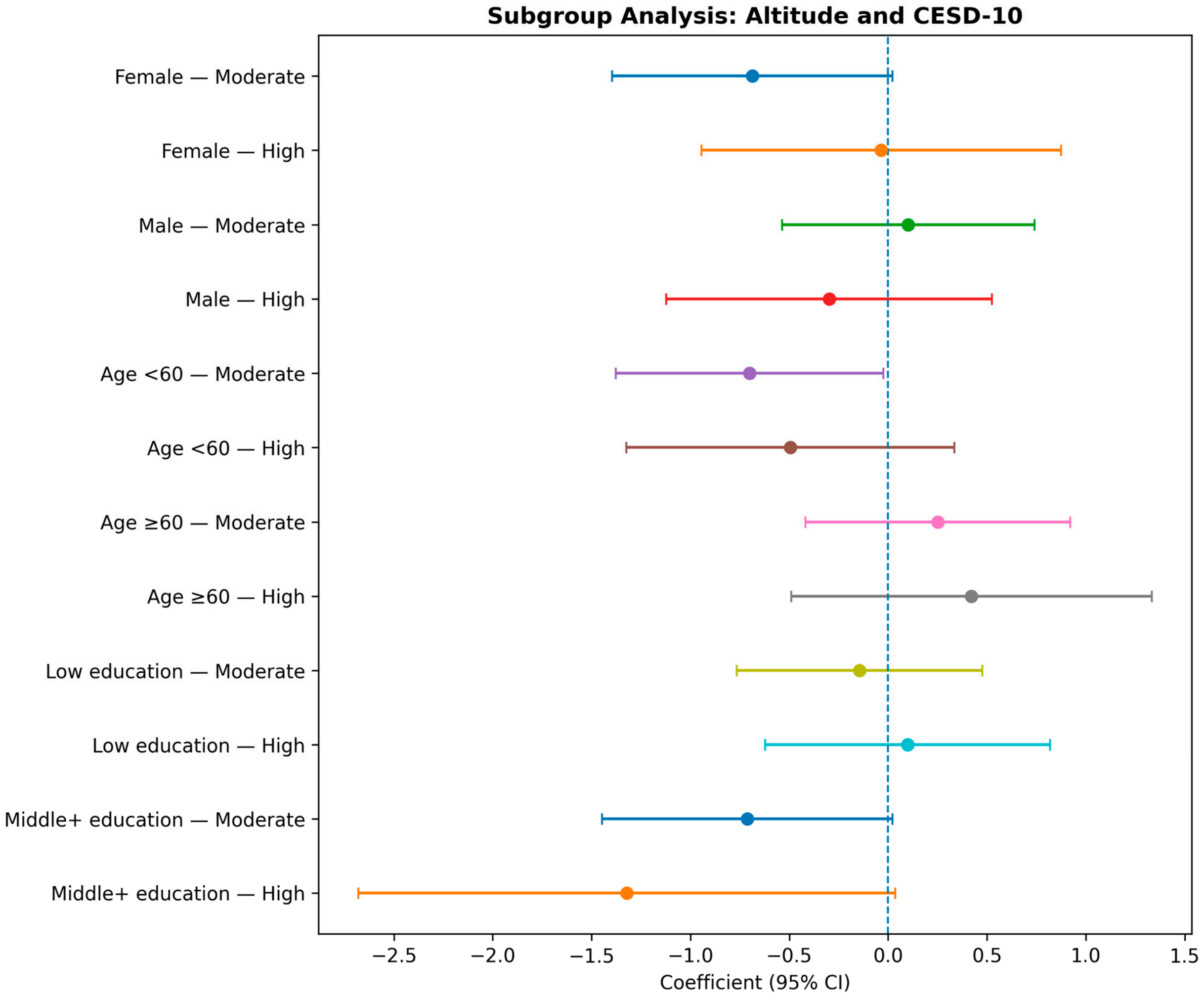

Figure S5. Subgroup-specific associations between altitude group and CESD-10 score. Estimates are exploratory; no formal between-subgroup interaction tests were reported.

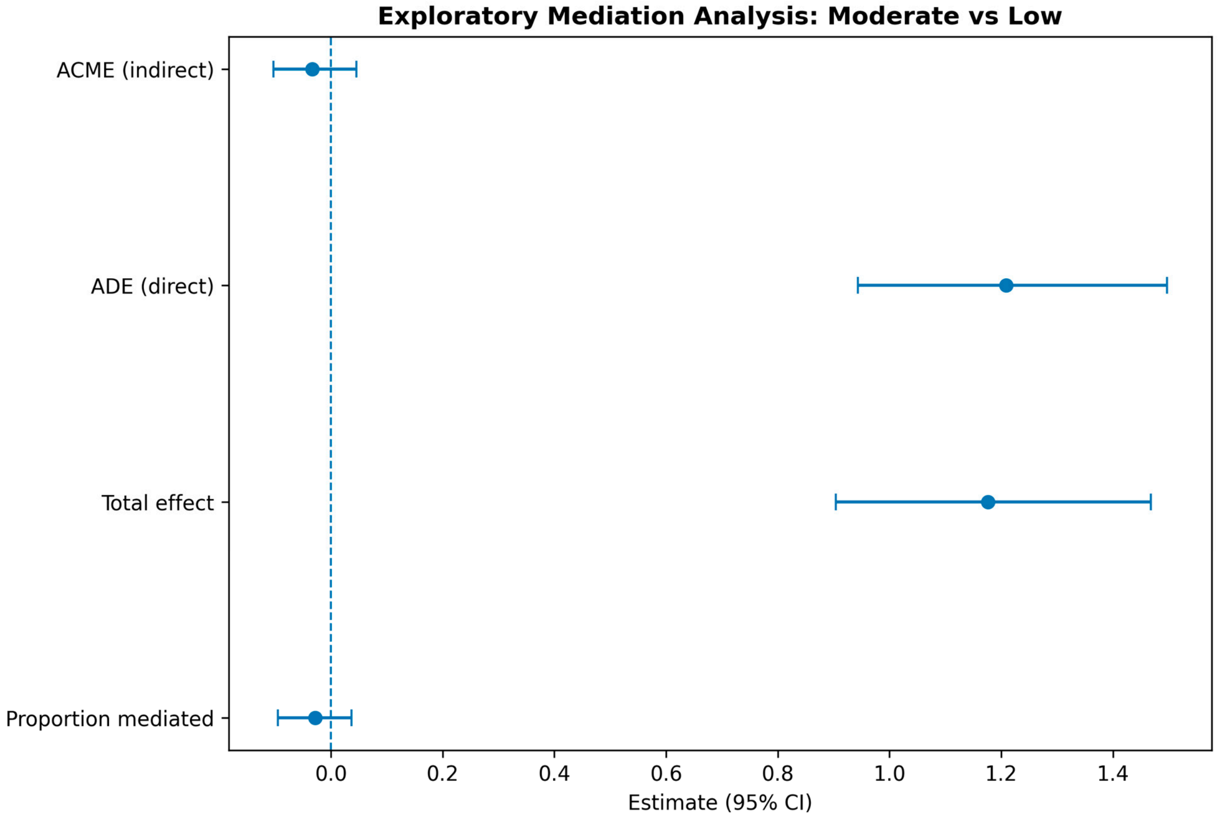

Figure S6. Exploratory mediation analysis comparing moderate with low altitude. ACME denotes the indirect effect through continuous sleep duration and ADE denotes the direct effect.

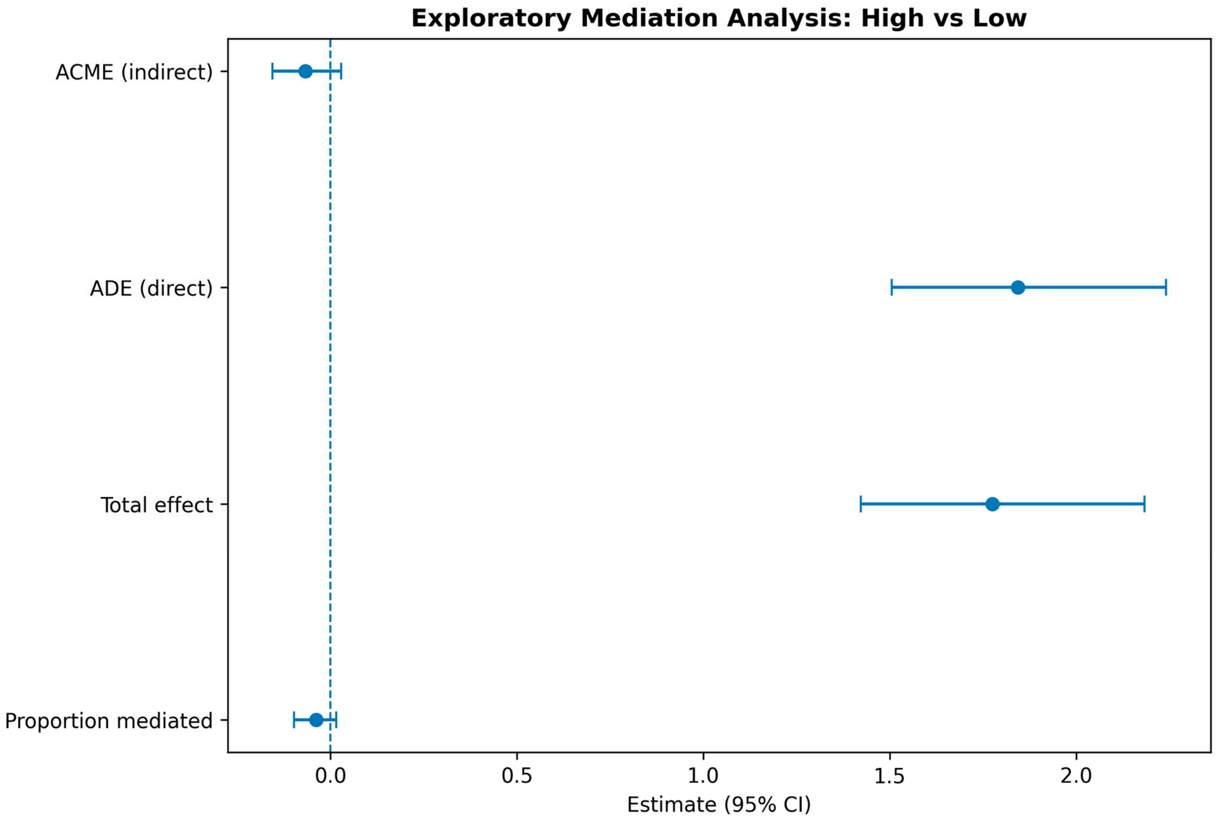

Figure S7. Exploratory mediation analysis comparing high with low altitude. ACME denotes the indirect effect through continuous sleep duration and ADE denotes the direct effect.

Among the fixed final analytical cohort of 15,562 participants, 13,870 had complete responses to all 10 CESD items. Cronbach's α = 0.802 (standardized α = 0.814), indicating good internal consistency in the current analytical sample.

Figure S8. Residual-versus-fitted plot for the fully adjusted primary linear regression model.

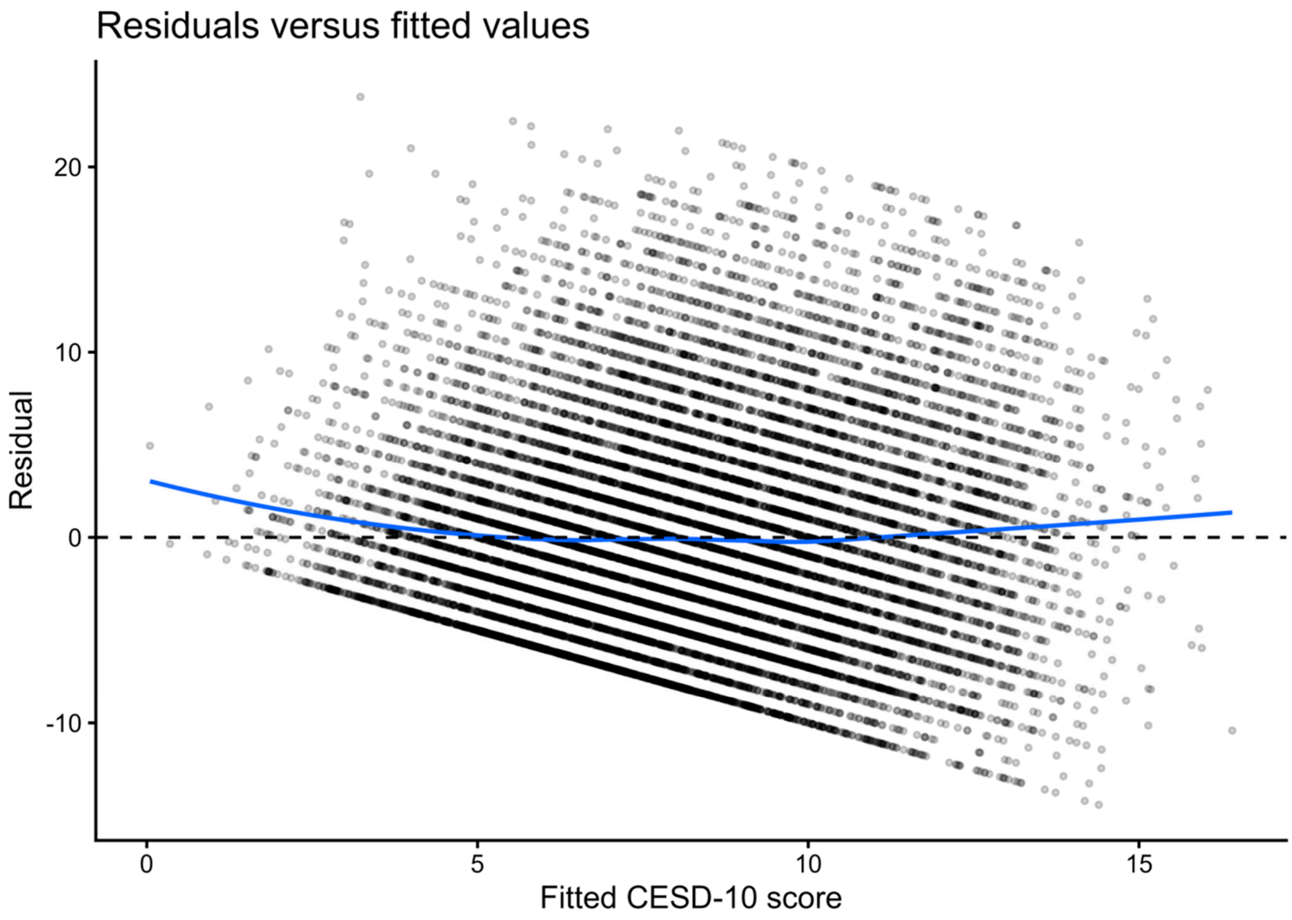

Figure S9. Normal Q-Q plot of residuals from the fully adjusted primary linear regression model.

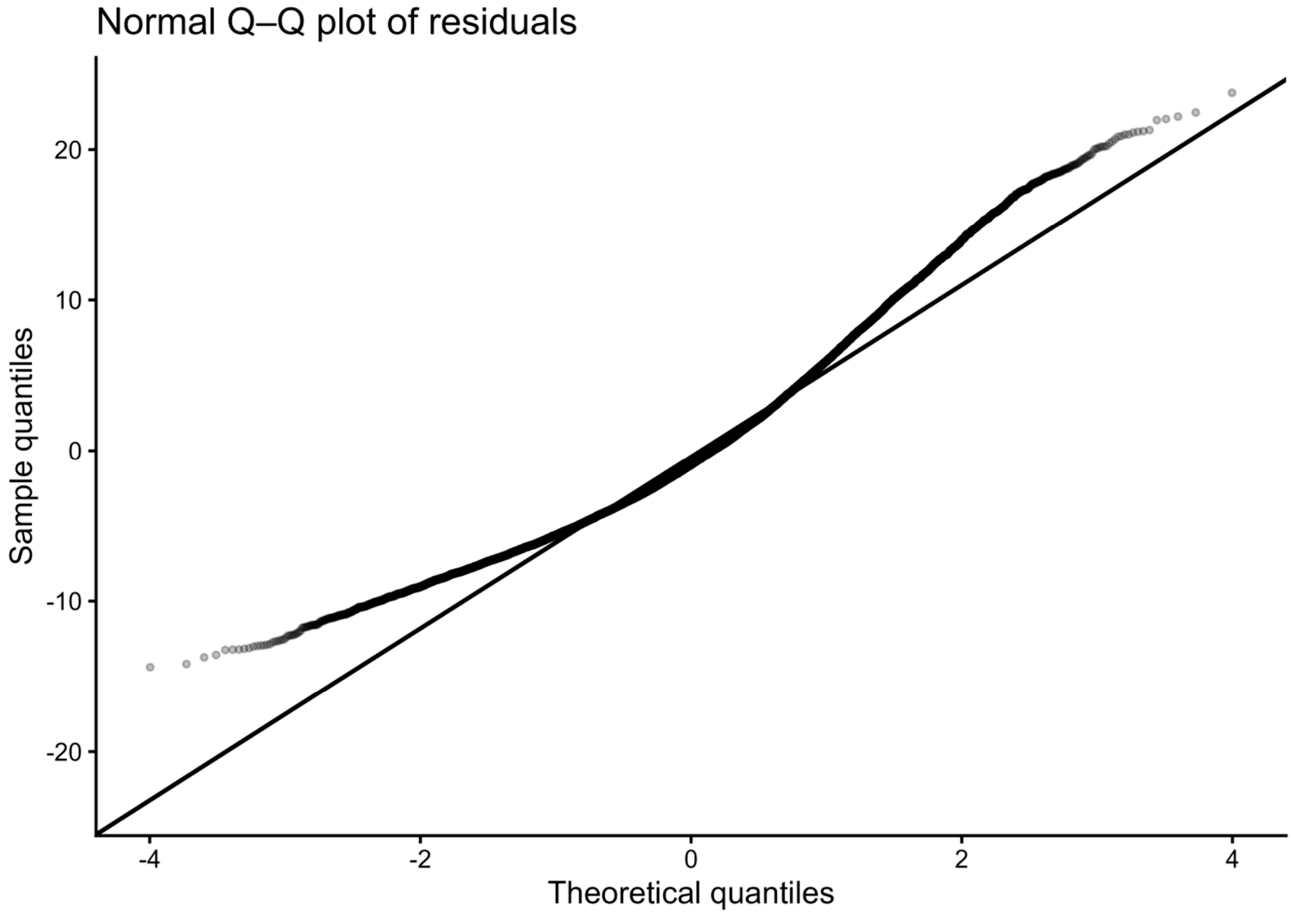

Abbreviations: CESD-10, 10-item Center for Epidemiologic Studies Depression Scale; CI, confidence interval; GAM, generalized additive model; REML, restricted maximum likelihood; edf, effective degrees of freedom.
